# Supplementary material for: Novel Co-Polyamides Containing Pendant Phenyl/Pyridinyl Groups with Potential Application in Water Desalination Processes
Source: Polymers (Basel). 2025 Jan 15;17(2):208. doi: 10.3390/polym17020208 (PMC11768725; doi:10.3390/polym17020208)
Supplement: Supplementary file 1 [file polymers-17-00208-s001.zip › polymers-3381787-supplementary.pdf]

Supplementary Materials

Novel co-polyamides containing pendant phenyl/pyridinyl groups with potential application in water desalination processes

Carolina Arriaza-Echanes[1], Claudio A. Terraza[2,3], Alain Tundidor-Camba[4], Loreto Sanhueza Ch.[5], and Pablo A. Ortiz[1,6]*.

[1] Centro de Nanotecnología Aplicada, Facultad de Ciencias, Ingeniería y Tecnología, Universidad Mayor, Camino La Pirámide 5750, 8580745, Huechuraba, Santiago, RM, Chile.

[2] Research Laboratory for Organic Polymers (RLOP), Department of Organic Chemistry, Pontificia Universidad Católica de Chile, 7820436, Santiago, RM, Chile.

[3] UC Energy Research Center, Pontificia Universidad Católica de Chile, 7820436, Santiago, RM, Chile.

[4] Department of Chemical & Biological Engineering, The University of Alabama, Tuscaloosa, AL 35487-0203, USA.

[5] Núcleo de Química y Bioquímica, Facultad de Ciencias, Ingeniería y Tecnología, Universidad Mayor, Camino La Pirámide 5750, 8580745, Huechuraba, Santiago, RM, Chile.

[6] Escuela de Ingeniería en Medio Ambiente y Sustentabilidad, Facultad de Ciencias, Ingeniería y Tecnología, Universidad Mayor, Camino La Pirámide 5750, 8580745, Huechuraba, Santiago, RM, Chile.

Synthetic equipment setup

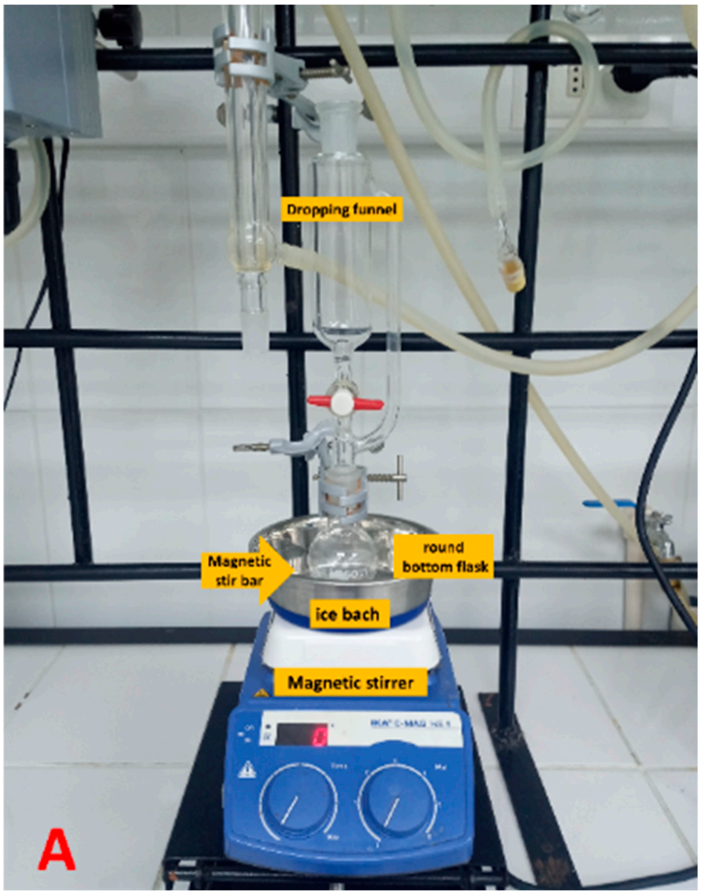

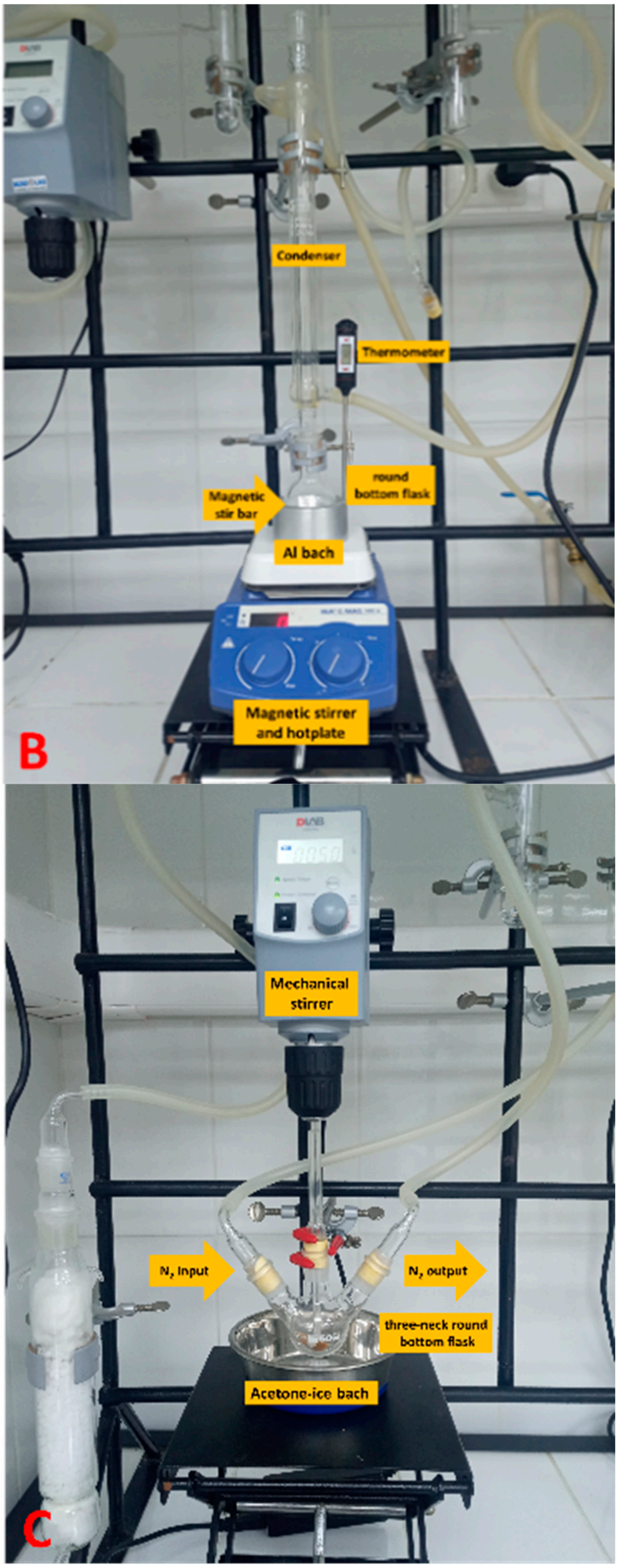

Figure S1. Experimental assembly for synthesis of dinitro (A), diamine (B) and polymers (C).

Spectroscopic data

3,5-Dinitro-*N*-phenylbenzamide (PhDN)

Yield: 96%. Mp: 239.0 – 240.0 °C. FT-IR-ATR (ZnSe, cm^{-1}): 3288 (N-H); 3198, 3131, 3104 (C-H, arom.); 1649 (C=O); 1632, 1595 (C=C); 1530 (N=O, asym), 1333, 1321 (N=O, sym); 920, 911, 757, 729, 715 (arom. *tri*-subst.); 688 (arom. *mono*-subst.). ^{1}H NMR (DMSO-d_6, δ, ppm): 10.78 (s, 1H, **6**); 9.15 (d, *J* = 2.0 Hz, 2H, **3**); 8.96 (t, *J* = 1.9 Hz, 1H, **1**); 7.75 (d, *J* = 8.0 Hz, 2H, **8**); 7.38 (t, *J* = 7.9 Hz, 2H, **9**); 7.15 (t, *J* = 7.9 Hz, 1H, **10**). ^{13}C NMR (DMSO-d_6, δ, ppm): 161.12 (**5**); 148.08 (**2**); 138.26 (**7**); 137.39 (**4**); 128.73 (**3**); 127.98 (**9**); 124.52 (**1**); 121.03 (**10**); 120.65 (**8**).

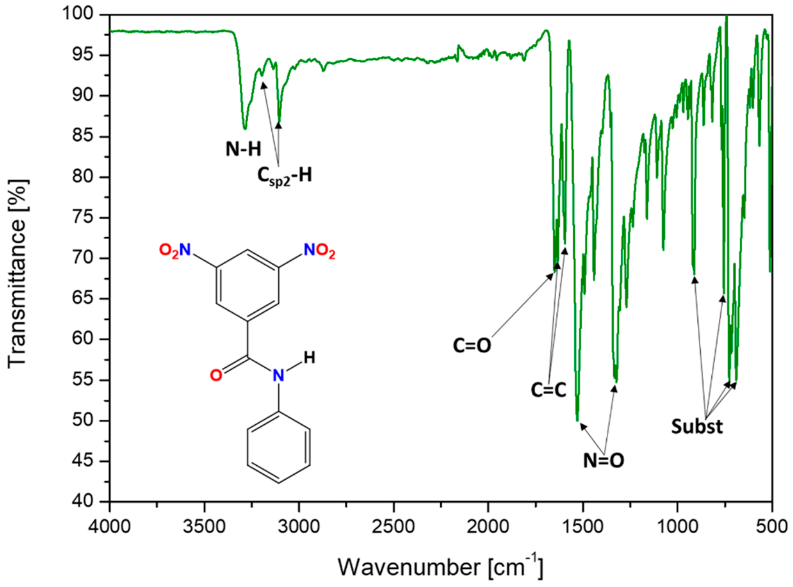

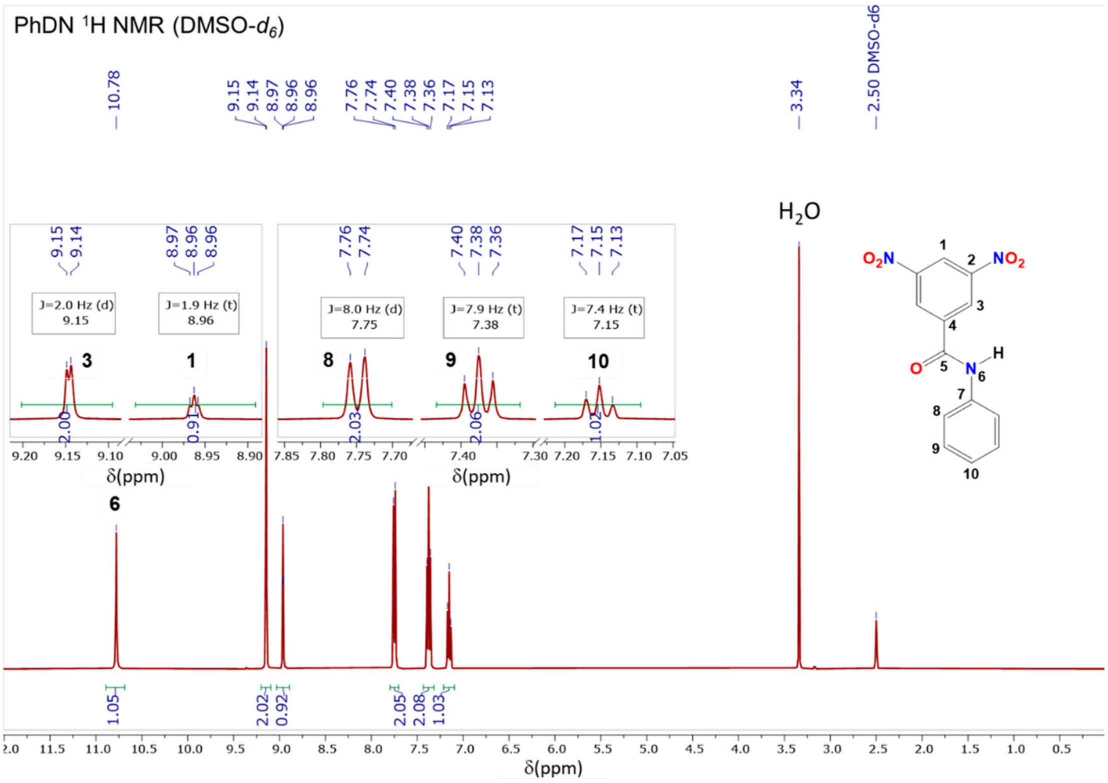
PhDN ^{1}H NMR (DMSO-d_6)
10.78
9.15 9.14 8.97 8.96 8.96
7.76 7.74 7.40 7.38 7.36 7.17 7.15 7.13
3.34
2.50 DMSO-d6
H$_2$O
J=2.0 Hz (d) 9.15
J=1.9 Hz (t) 8.96
J=8.0 Hz (d) 7.75
J=7.9 Hz (t) 7.38
J=7.4 Hz (t) 7.15
2.00 0.91 2.03 2.06 1.02
1.05 2.02 0.92 2.05 2.08 1.03
δ(ppm)

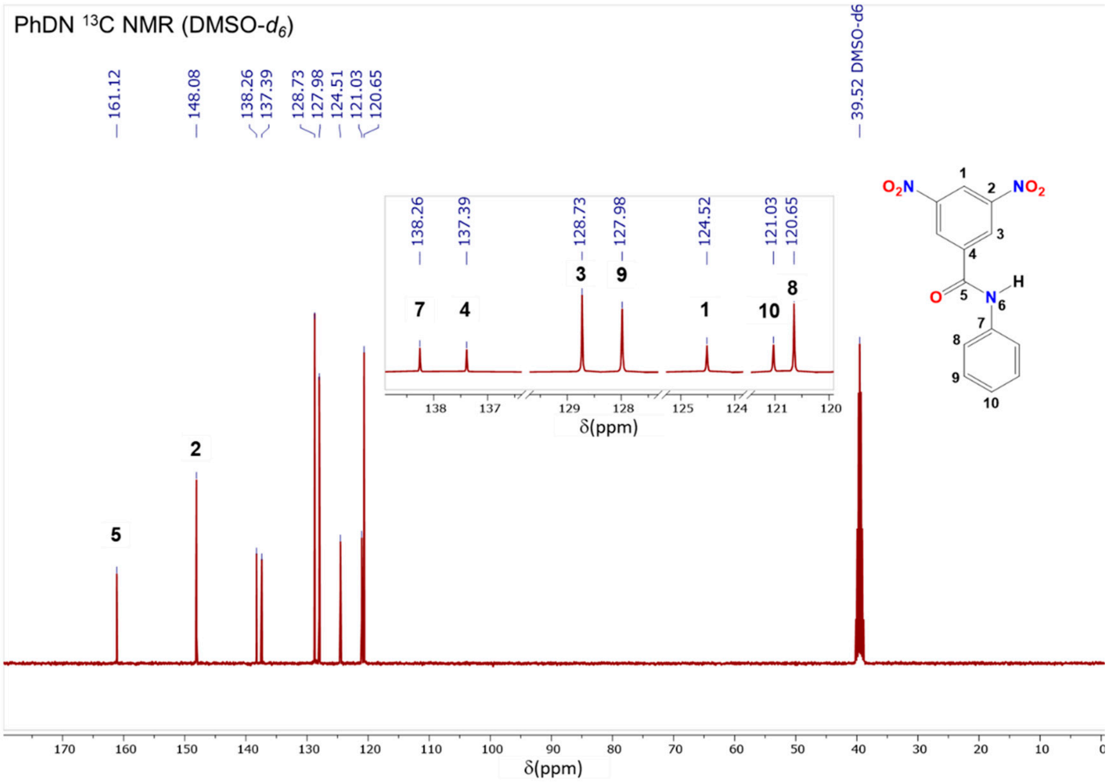
PhDN ^{13}C NMR (DMSO-d_6)
161.12
148.08
138.26 137.39
128.73 127.98 124.51 121.03 120.65
39.52 DMSO-d6
138.26 137.39 128.73 127.98 124.52 121.03 120.65
δ(ppm)

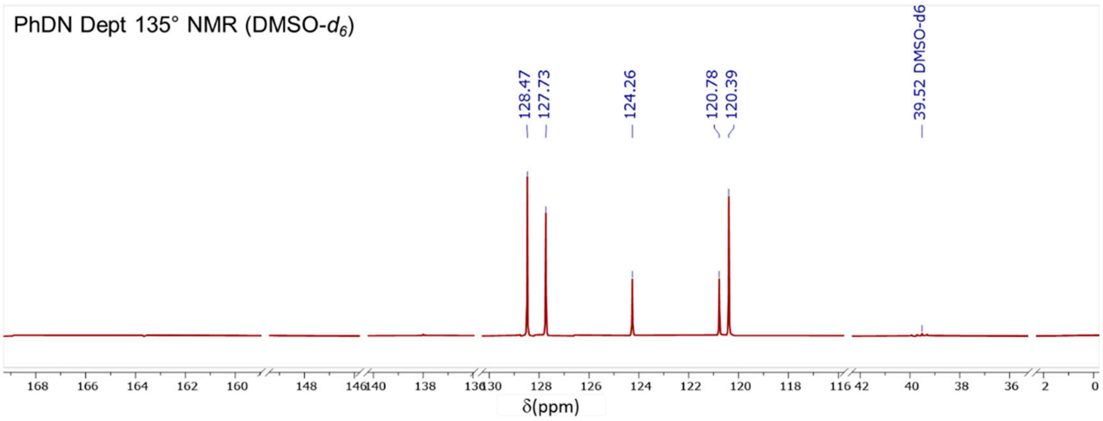

Figure S2. Spectroscopy characterization of 3,5-dinitro-*N*-phenylbenzamide.

3,5-Dinitro-*N*-(pyridin-4-yl)benzamide (PyDN)

Yield: 80%. Mp: 262.0 – 272.0 °C. FT-IR-ATR (ZnSe, cm^{-1}): 3096 (N-H); 3077, 3059, 3040 (C-H, arom.); 1676 (C=O); 1627 (C=N); 1596, 1516 (C=C); 1534 (N=O, asym); 1346, 1334 (N=O, sym); 921, 733, 724, 713 (arom. *tri*-subst); 830, 819 (arom. *mono*-subst). ^{1}H NMR (DMSO-*d6*, δ, ppm): 11.15 (s, 1H, **6**); 9.16 (s, 2H, **3**); 9.02 (s, 1H, **1**); 8.54 (d, *J* = 3.1 Hz, 2H, **9**); 7.79 (d, *J* = 5.0 Hz, 2H, **8**). ^{13}C NMR (DMSO-*d6*, δ, ppm): 162.31 (**5**); 150.42 (**9**); 148.12 (**2**); 145.25 (**7**); 136.76 (**4**); 128.23 (**3**); 121.52 (**1**); 114.33 (**8**).

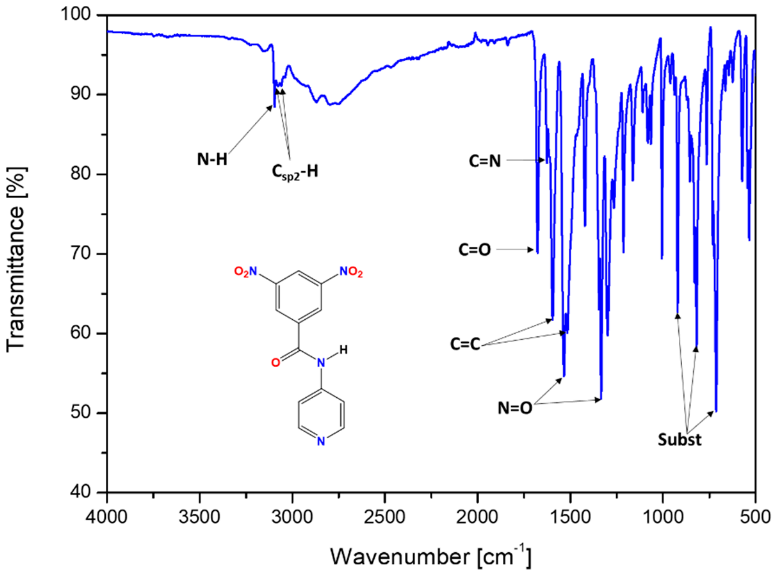

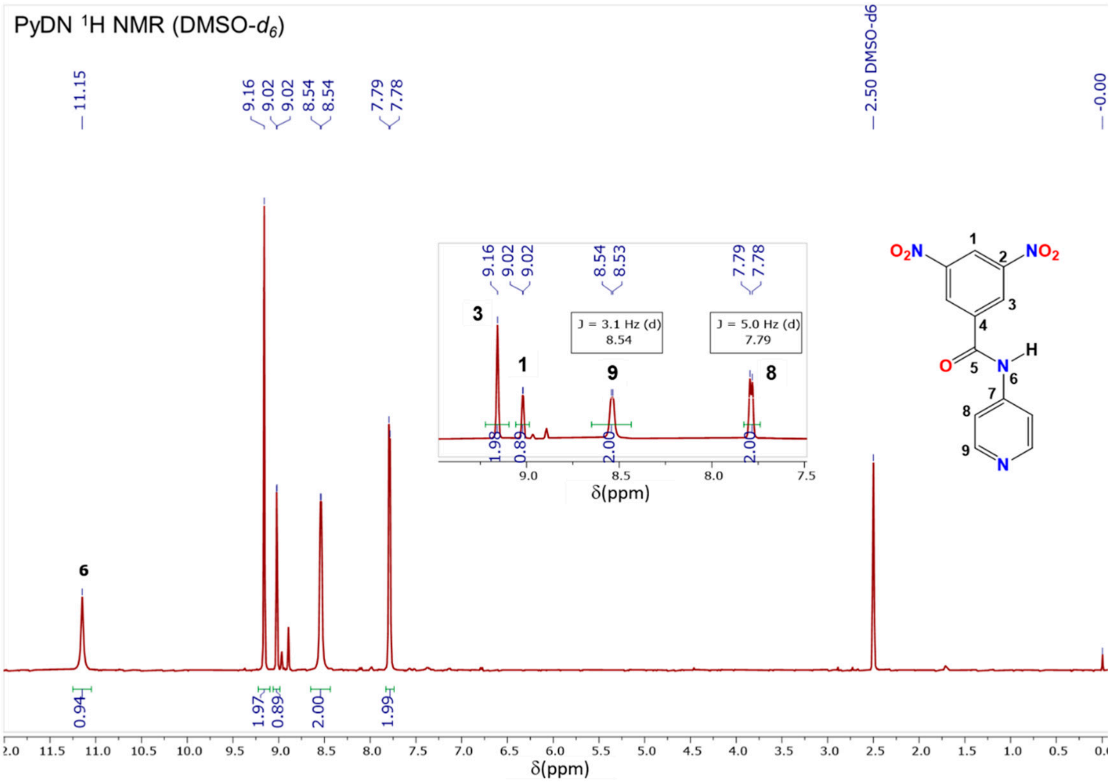

PyDN ¹H NMR (DMSO-d6)
11.15
9.16
9.02
9.02
8.54
8.54
7.79
7.78
2.50 DMSO-d6
-0.00
J = 3.1 Hz (d) 8.54
J = 5.0 Hz (d) 7.79
O2N
NO2
H
N
O
0.94
1.97
0.89
2.00
1.99
1.98
δ(ppm)

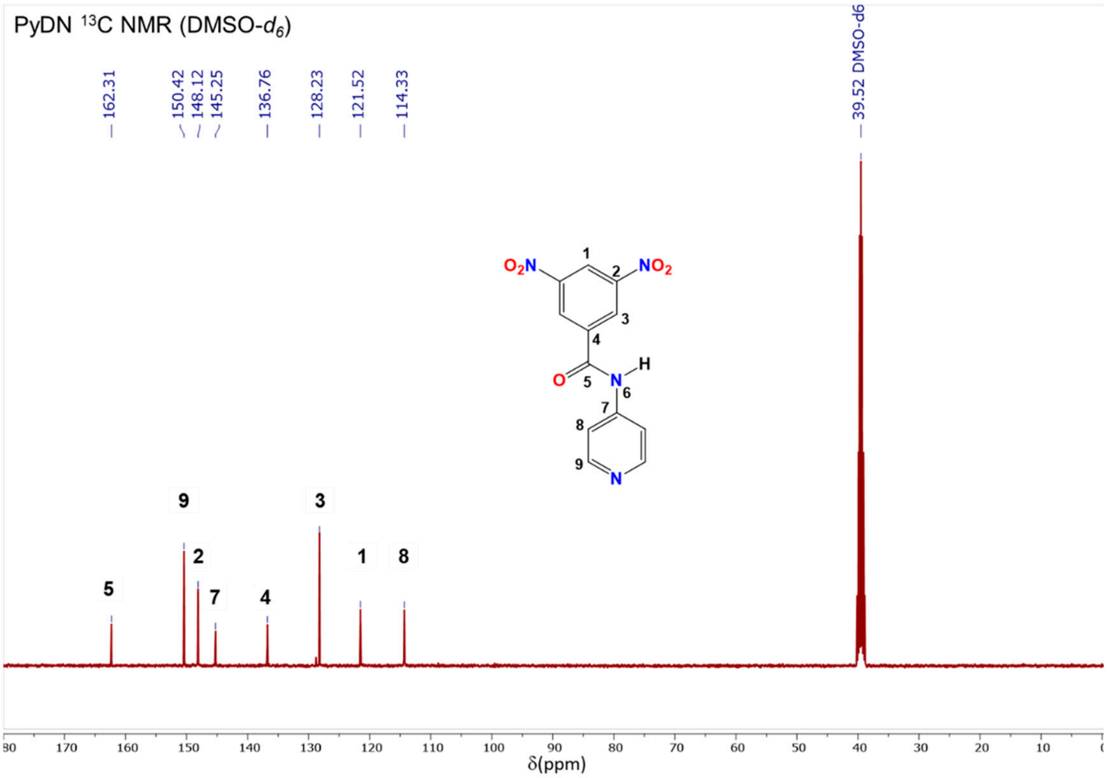

PyDN ¹³C NMR (DMSO-d6)
162.31
150.42
148.12
145.25
136.76
128.23
121.52
114.33
39.52 DMSO-d6
O2N
NO2
H
N
O
δ(ppm)

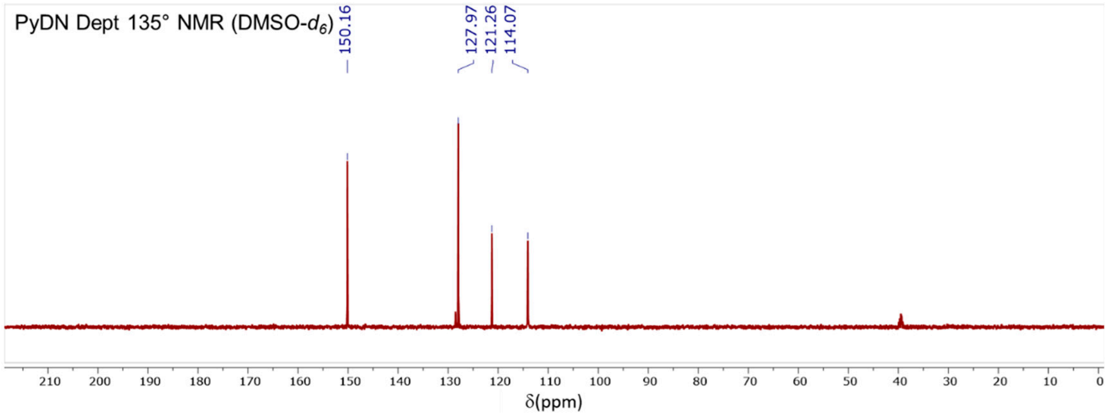

Figure S3. Spectroscopy characterization of 3,5-dinitro-*N*-(pyridin-4-yl)benzamide.

3,5-Dinitro-*N*-(pyridin-4-ylmethyl)benzamide (PyMDN)

Yield: 85%. Mp: 200.0 – 201.0 °C. FT-IR-ATR (ZnSe, cm^{-1}): 3322 (N-H); 3112, 3050, 3038 (C-H, arom.); 2943, 2864 (C-H, aliph); 1675, 1649 (C=O); 1627 (C=N), 1604, 1500 (C=C); 1533 (N=O, asym); 1341 (N=O, sym); 909, 831, 792, 730, 719, 708 (arom. *tri*-subst); 709 (arom. *mono*-subst). ^{1}H NMR (DMSO-d_6, δ, ppm): 9.82 (t, J = 5.1 Hz, 1H, **6**); 9.11 (s, 2H, **3**); 8.99 (s, 1H, **1**); 8.53 (d, J = 4.5 Hz, 2H, **10**); 7.37 (d, J = 4.6 Hz, 2H, **9**); 4.59 (d, J = 5.6 Hz, 2H, **7**). ^{13}C NMR (DMSO-d_6, δ, ppm): 162.5 (**5**); 149.6 (**10**); 148.2 (**2**); 147.6 (**8**); 136.5 (**4**); 127.6 (**3**); 122.3 (**9**); 121.0 (**1**); 42.2 (**7**).

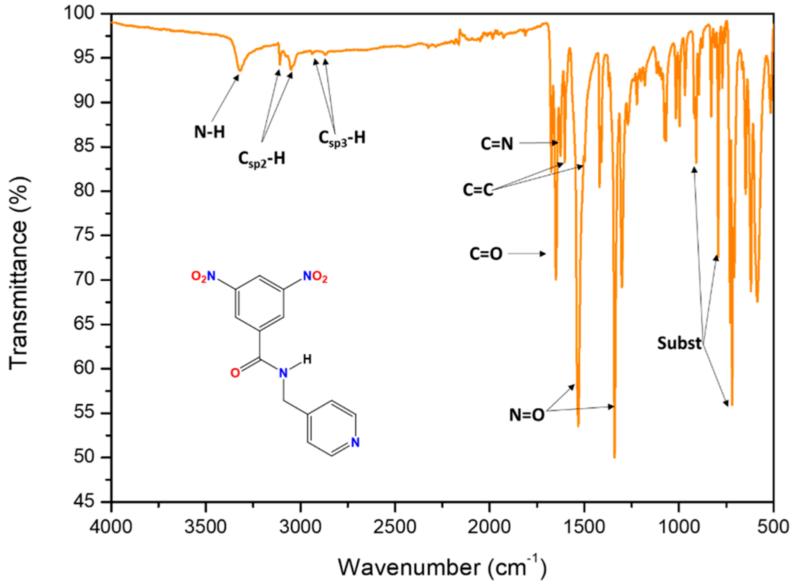

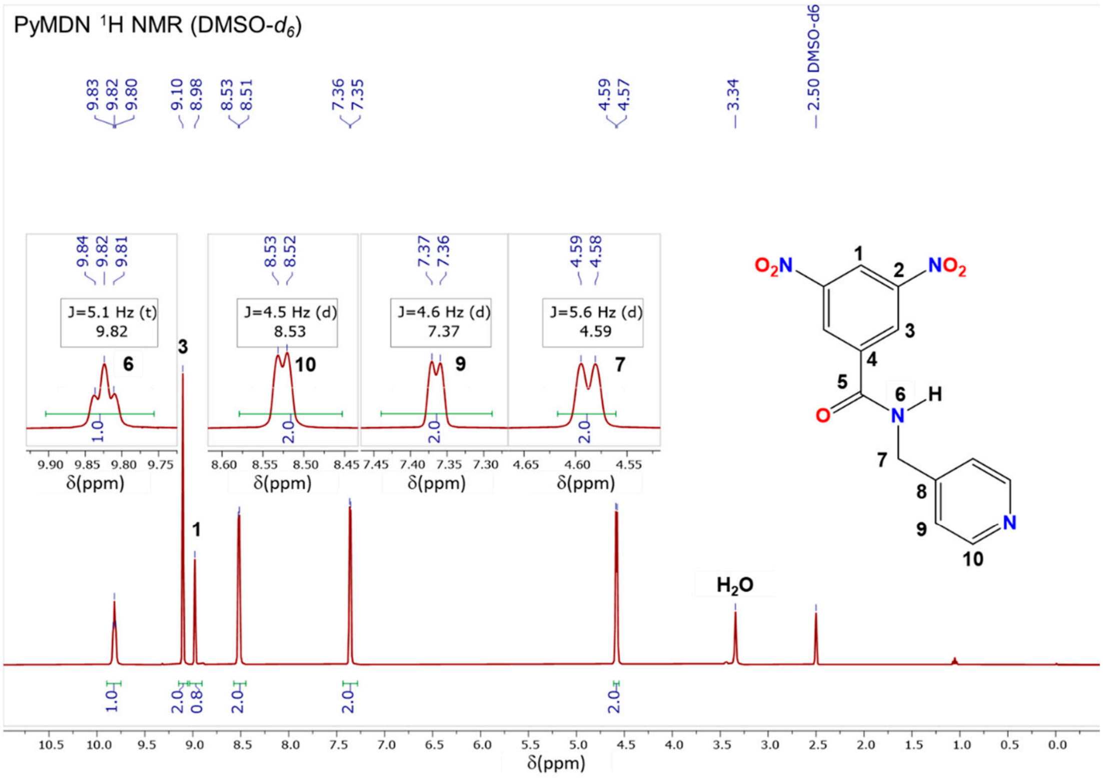

PyMDN ¹H NMR (DMSO-d6)
9.83
9.82
9.80
9.10
8.98
8.53
8.51
7.36
7.35
4.59
4.57
3.34
2.50 DMSO-d6
9.84
9.82
9.81
J=5.1 Hz (t)
9.82
6
8.53
8.52
J=4.5 Hz (d)
8.53
10
7.37
7.36
J=4.6 Hz (d)
7.37
9
4.59
4.58
J=5.6 Hz (d)
4.59
7
1.0
2.0
2.0
2.0
δ(ppm)
3
1
H2O
O2N
NO2
1
2
3
4
5
6
H
O
N
7
8
9
N
10
1.0
2.0
0.8
2.0
2.0
2.0
δ(ppm)

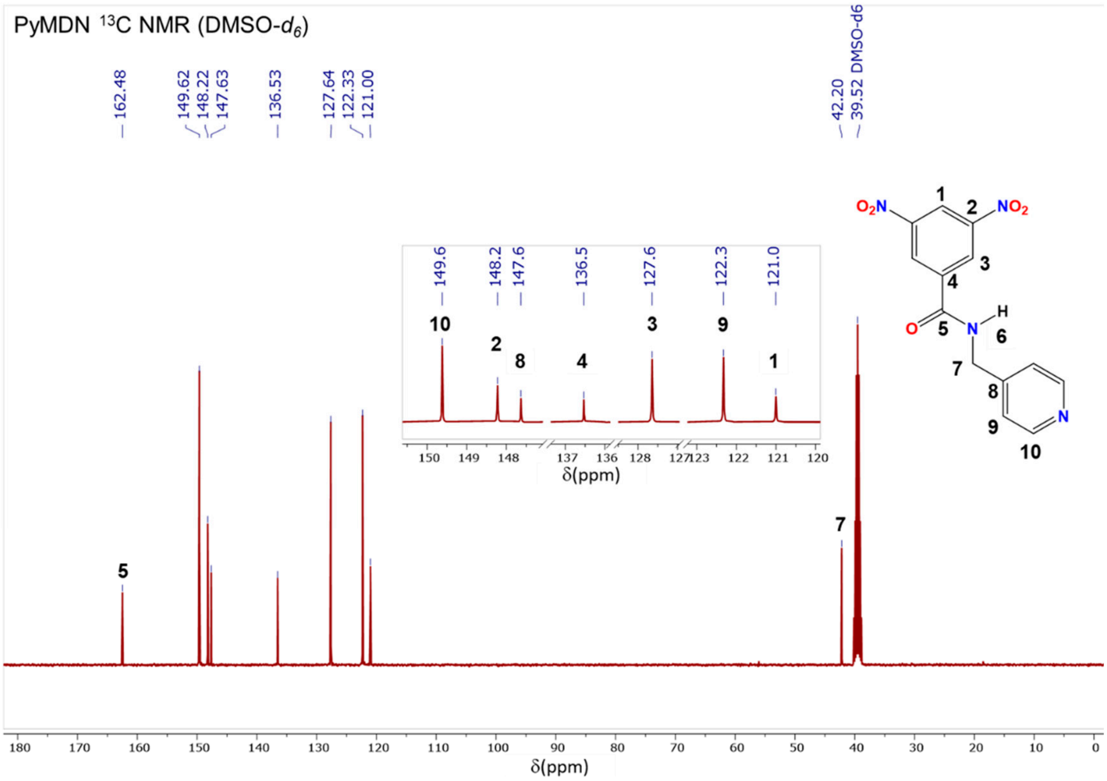

PyMDN ¹³C NMR (DMSO-d6)
162.48
149.62
148.22
147.63
136.53
127.64
122.33
121.00
42.20
39.52 DMSO-d6
149.6
148.2
147.6
136.5
127.6
122.3
121.0
10
2
8
4
3
9
1
δ(ppm)
O2N
NO2
1
2
3
4
5
6
H
O
N
7
8
9
N
10
5
7
δ(ppm)

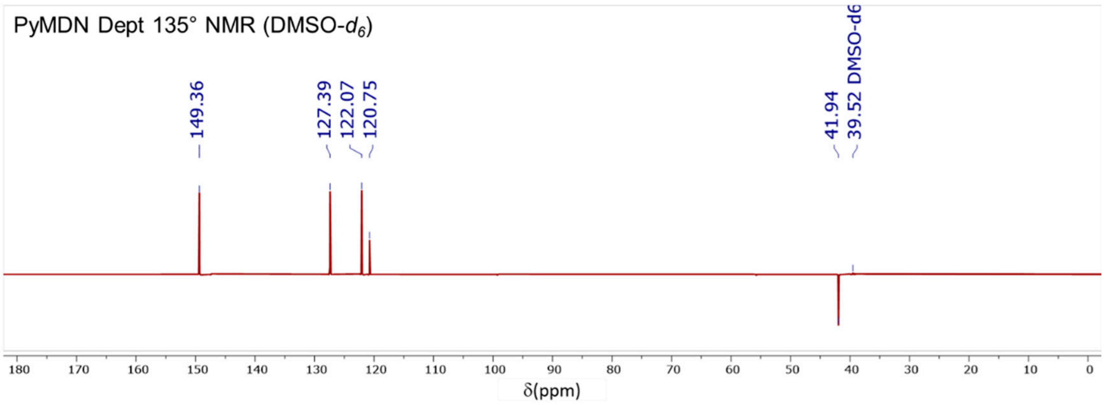

Figure S4. Spectroscopy characterization of 3,5-dinitro-*N*-(pyridin-4-ylmethyl) benzamide.

3,5-Diamino-*N*-phenylbenzamide (PhDA)

Yield: 85%. Mp: 191.0 – 191.7 °C. FT-IR-ATR (ZnSe, cm^{-1}): 3385, 3312 (N-H, amine); 3278 (N-H, amide); 3127, 3104, 3047, 3014 (C-H, arom.); 1654 (C=O); 1592, 1529, 1501, 1490 (C=C); 851, 843, 789, 772, 747, 710 (arom. *tri*-subst); 680 (arom. *mono*-subst). ^1H NMR (DMSO-d_6, δ, ppm): 9.92 (d, J = 13.3 Hz, 1H, **6**); 7.72 (s, 2H, **8**); 7.30 (s, 2H, **9**); 7.05 (s, 1H, **10**); 6.31 (d, J = 13.5 Hz, 2H, **3**); 6.01 (d, J = 13.1 Hz, 2H, **3**); 4.95 (s, 4H, **11**). ^{13}C NMR (DMSO-d_6, δ, ppm): 167.29 (**5**); 149.09 (**2**); 139.60 (**7**); 136.88 (**4**); 128.49 (**9**); 123.13 (**10**); 120.00 (**8**); 102.37 (**1,3**).

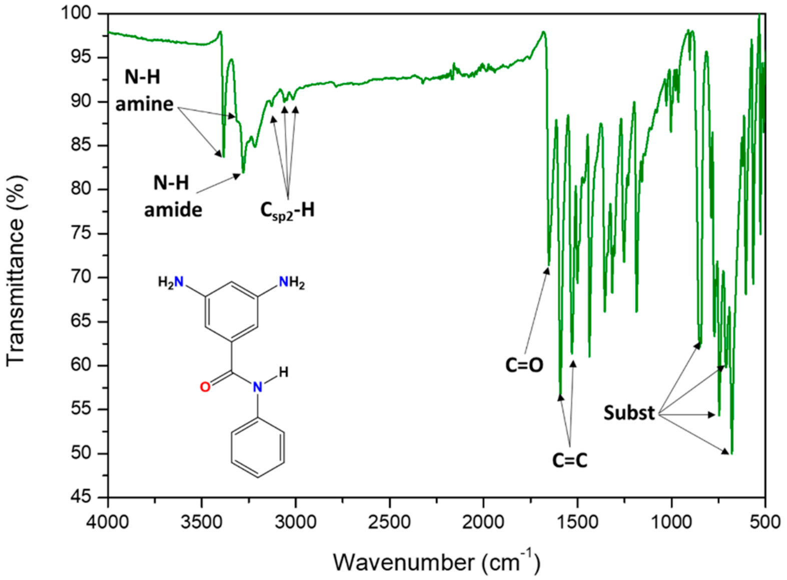

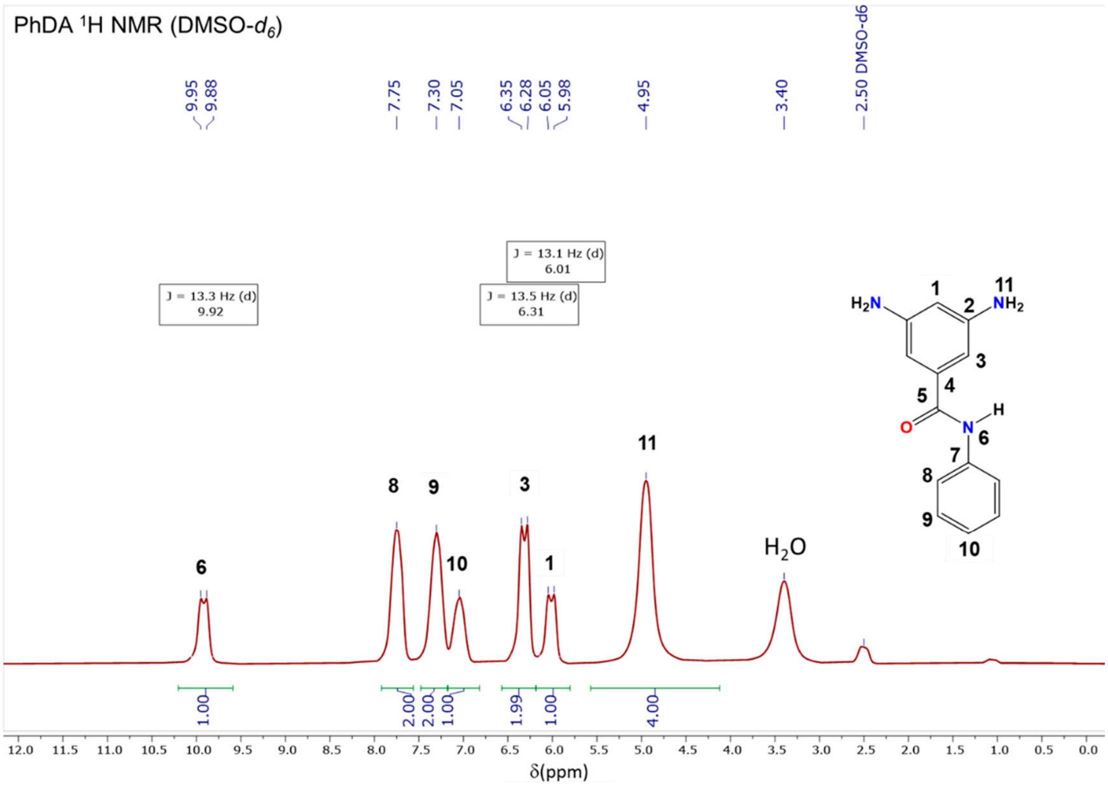

PhDA ¹H NMR (DMSO-d6)
9.95
9.88
7.75
7.30
7.05
6.35
6.28
6.05
5.98
4.95
3.40
2.50 DMSO-d6
J = 13.3 Hz (d) 9.92
J = 13.1 Hz (d) 6.01
J = 13.5 Hz (d) 6.31
H2N
NH2
O
N
H
1 2 3 4 5 6 7 8 9 10 11
H2O
1.00
2.00
2.00
1.00
1.99
1.00
4.00
12.0 11.5 11.0 10.5 10.0 9.5 9.0 8.5 8.0 7.5 7.0 6.5 6.0 5.5 5.0 4.5 4.0 3.5 3.0 2.5 2.0 1.5 1.0 0.5 0.0
δ(ppm)

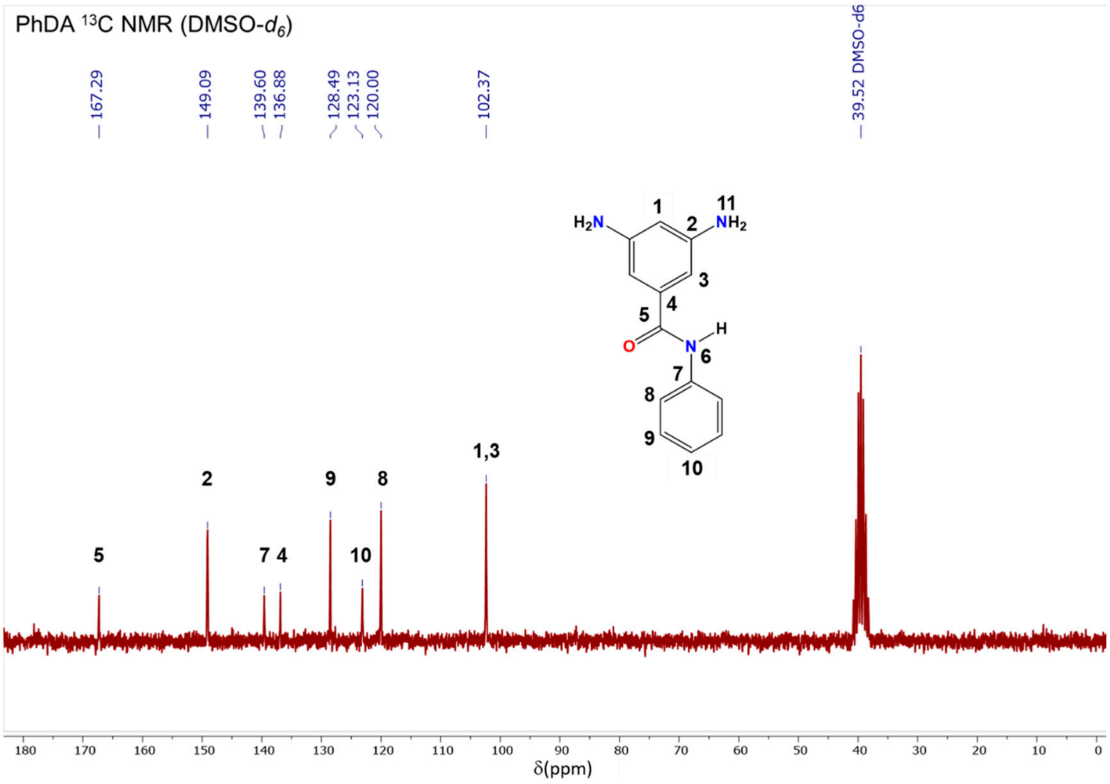

PhDA ¹³C NMR (DMSO-d6)
167.29
149.09
139.60
136.88
128.49
123.13
120.00
102.37
39.52 DMSO-d6
H2N
NH2
O
N
H
1 2 3 4 5 6 7 8 9 10 11
5
2
7 4
9
10
8
1,3
180 170 160 150 140 130 120 110 100 90 80 70 60 50 40 30 20 10 0
δ(ppm)

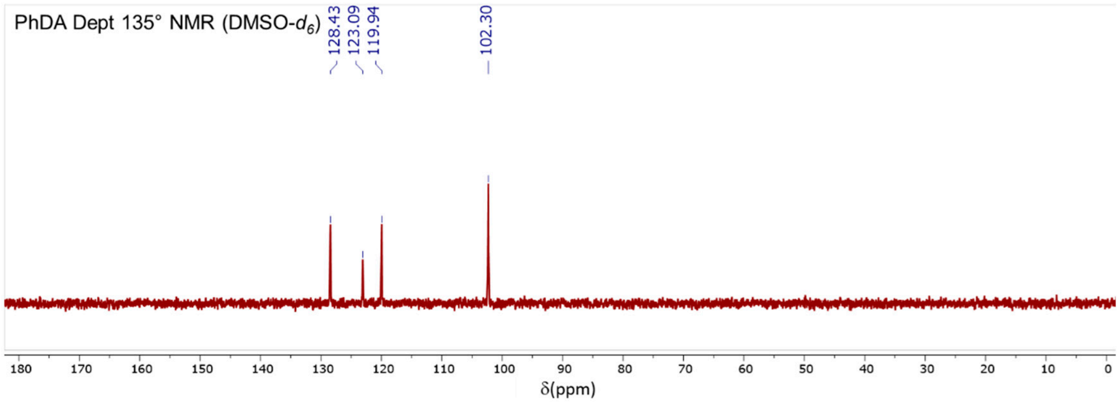

Figure S5. Spectroscopy characterization of 3,5-diamino-*N*-phenylbenzamide.

3,5-Diamino-*N*-(pyridin-4-yl)benzamide (PyDA)

Yield: 80%. Mp: 207.0 – 208.0 °C. FT-IR-ATR (ZnSe, cm^{-1}): 3399, 3317 (N-H, amine); 3179 (N-H, amide); 3057, 3040, 3021 (C-H, arom.); 1668 (C=O); 1622 (C=N); 1590, 1518 (C=C); 916, 759 (arom. *tri*-subst); 842, 820 (arom. *mono*-subst). ^1H NMR (DMSO-d_6, δ, ppm): 10.33 (s, 1H, **6**); 8.43 (d, *J* = 6.4 Hz, 2H, **9**); 7.76 (d, *J* = 6.4 Hz, 2H, **8**); 6.32 (d, *J* = 2.0 Hz, 2H, **3**); 6.05 (d, *J* = 2.0 Hz, 2H, **1**); 5.00 (s, 4H, **10**); ^{13}C NMR (DMSO-d_6, δ, ppm): 168.20 (**5**); 150.20 (**9**); 149.29 (**2**); 146.30 (**7**); 136.08 (**4**); 113.81 (**8**); 102.71 (**1**); 102.38 (**3**).

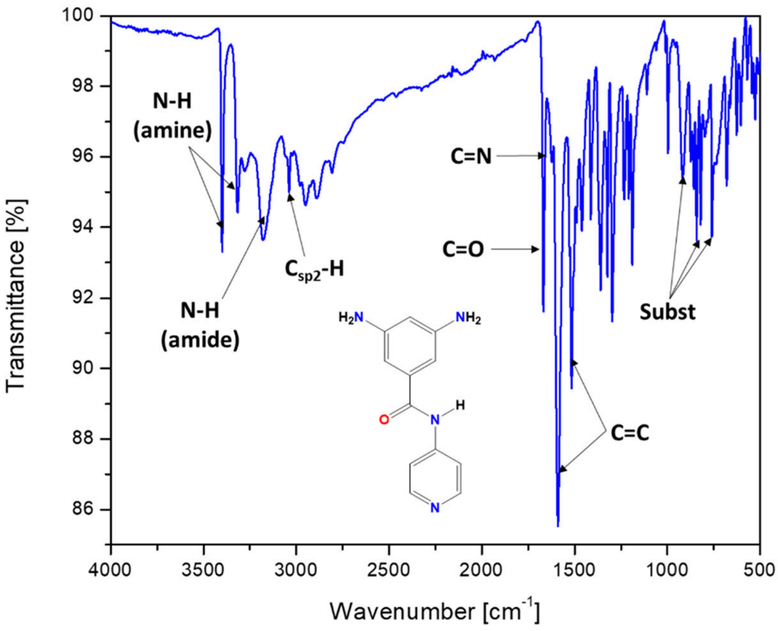

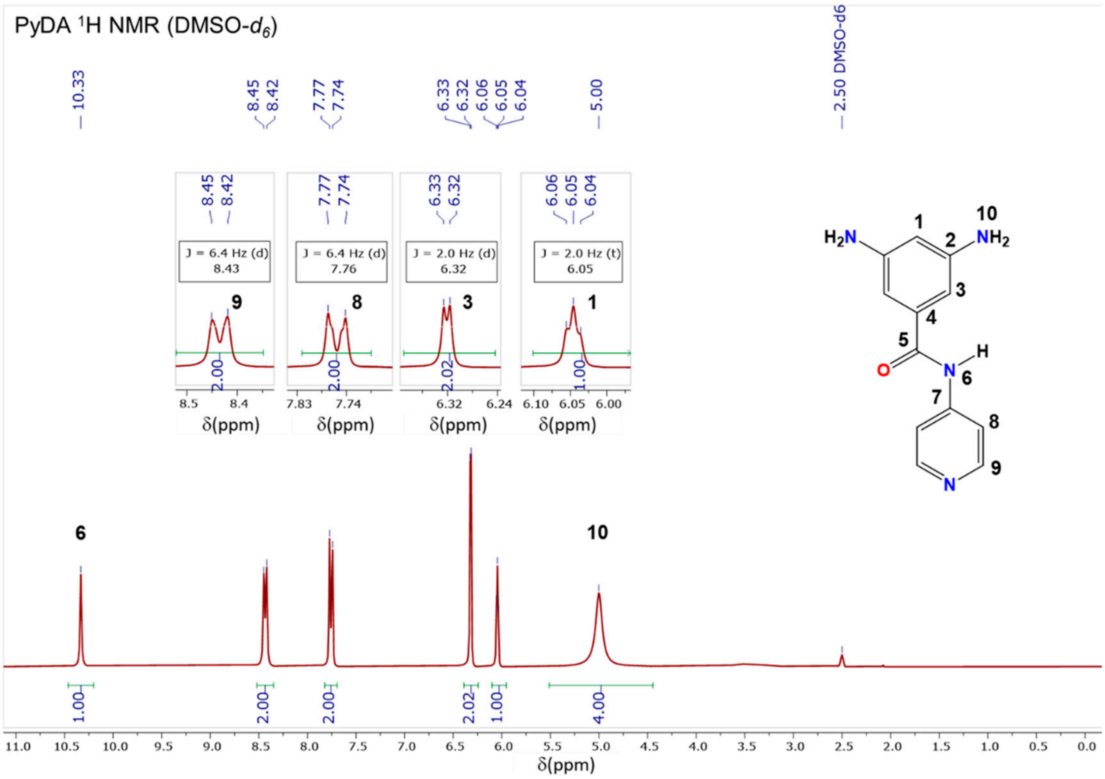
PyDA 1H NMR (DMSO-d6)
10.33
8.45
8.42
7.77
7.74
6.33
6.32
6.06
6.05
6.04
5.00
2.50 DMSO-d6
J = 6.4 Hz (d) 8.43
J = 6.4 Hz (d) 7.76
J = 2.0 Hz (d) 6.32
J = 2.0 Hz (t) 6.05
δ(ppm)

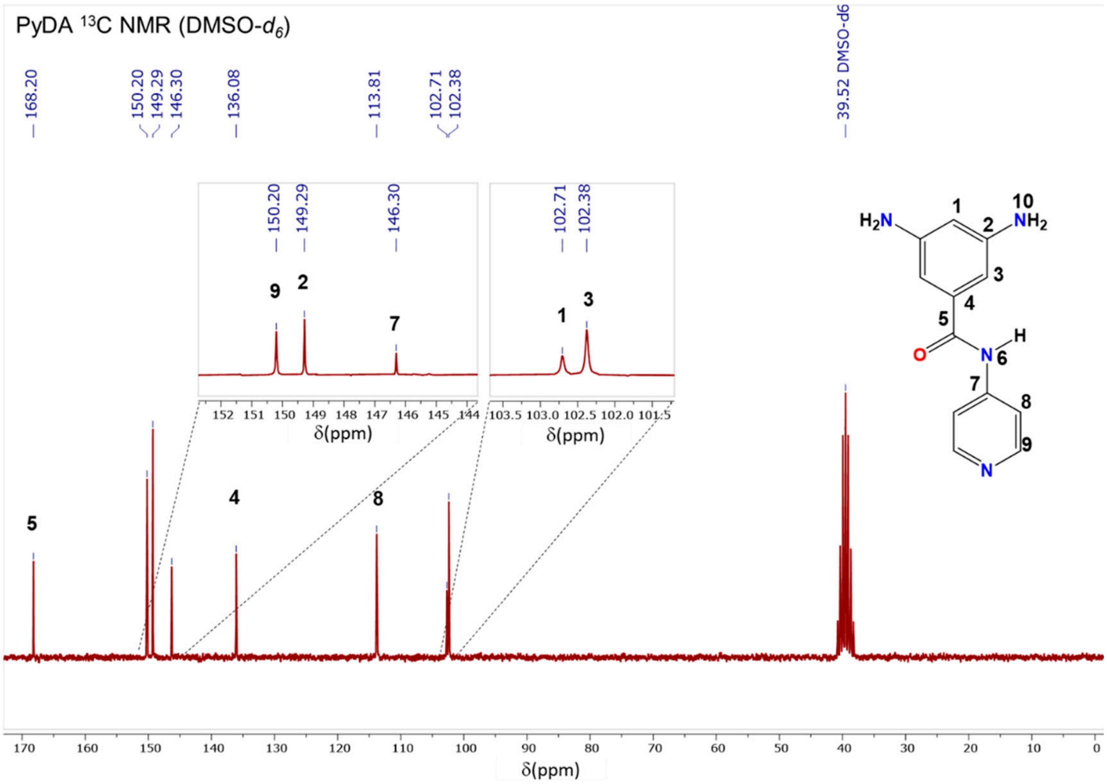
PyDA 13C NMR (DMSO-d6)
168.20
150.20
149.29
146.30
136.08
113.81
102.71
102.38
39.52 DMSO-d6
δ(ppm)

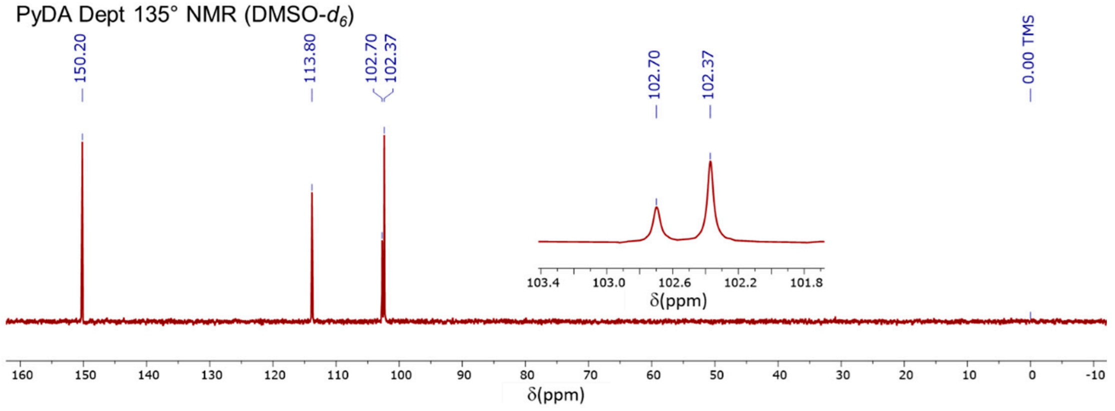

Figure S6. Spectroscopy characterization of 3,5-diamino-*N*-(pyridin-4-yl)benzamide.

3,5-Diamino-*N*-(pyridin-4-ylmethyl)benzamide (PyMDA)

Yield: 80%. Mp: 167 – 169 °C. FT-IR-ATR (ZnSe, cm^{-1}): 3412, 3339 (N-H, amine); 3204 (N-H, amide); 3072, 3048, 3024 (C-H, arom.); 2994, 2974, 2924 (C-H, aliph); 1632, 1651 (C=O); 1595, 1563, 1538, 1488 (C=C); 909, 845, 801, 769, 739, 708 (arom. *tri*-subst); 681 (arom. *mono*-subst). ^{1}H NMR (DMSO-*d₆*, δ, ppm): 8.67 (t, *J* = 5.7 Hz, 1H, **6**); 8.49 (d, *J* = 4.8 Hz, 2H, **10**); 7.26 (d, *J* = 4.8 Hz, 2H, **9**); 6.30 (s, 2H, **3**); 5.99 (s, 1H, **1**); 4.89 (s, 4H, **11**); 4.41 (d, *J* = 5.9 Hz, 2H, **7**). ^{13}C NMR (DMSO-d_6, δ, ppm): 168.48 (**5**); 149.46 (**10**); 149.15 (**2**); 149.09 (**8**); 135.97 (**4**); 122.11 (**9**); 102.28 (**1**); 102.13 (**3**); 41.59 (**7**).

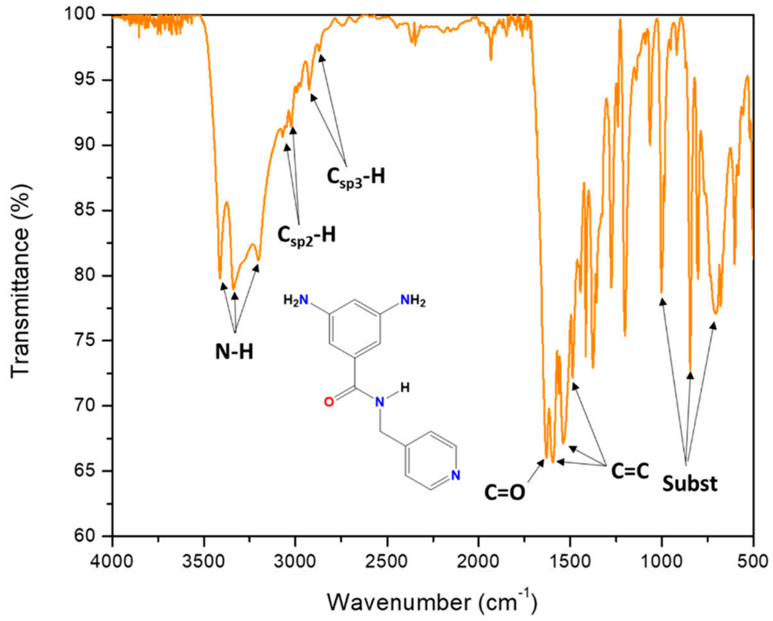

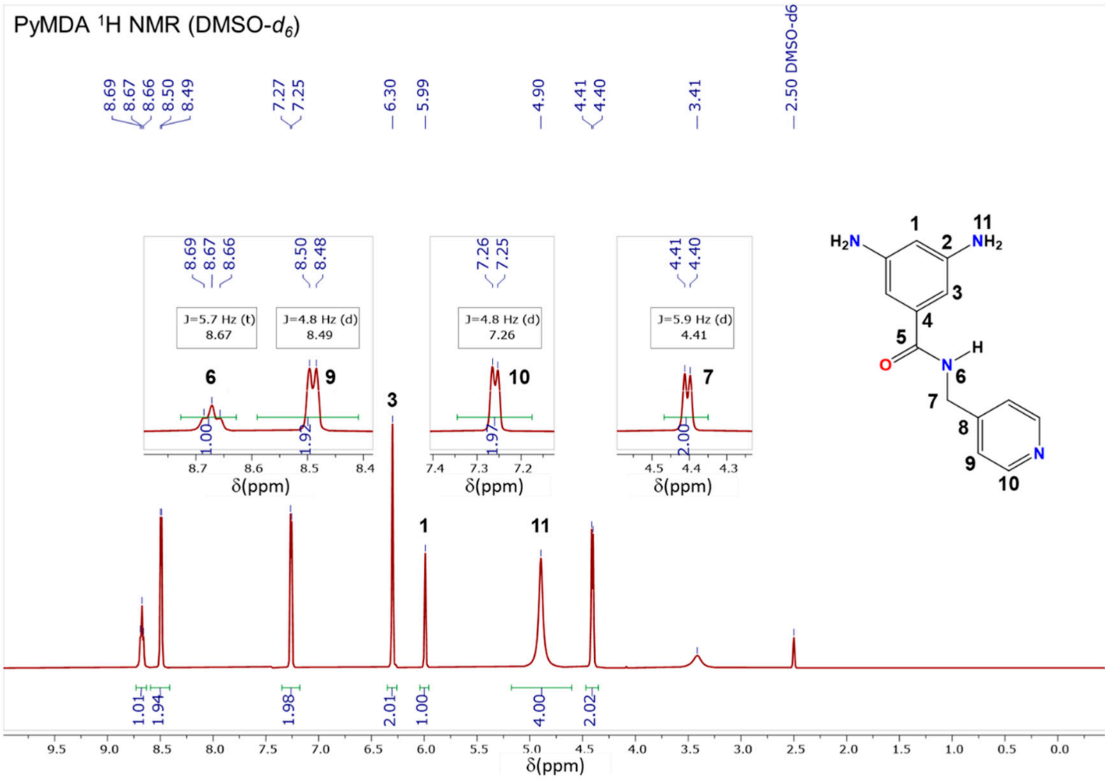
PyMDA ¹H NMR (DMSO-d6)
8.69 8.67 8.66 8.50 8.49 7.27 7.25 6.30 5.99 4.90 4.41 4.40 3.41 2.50 DMSO-d6
J=5.7 Hz (t) 8.67
J=4.8 Hz (d) 8.49
J=4.8 Hz (d) 7.26
J=5.9 Hz (d) 4.41
δ(ppm)

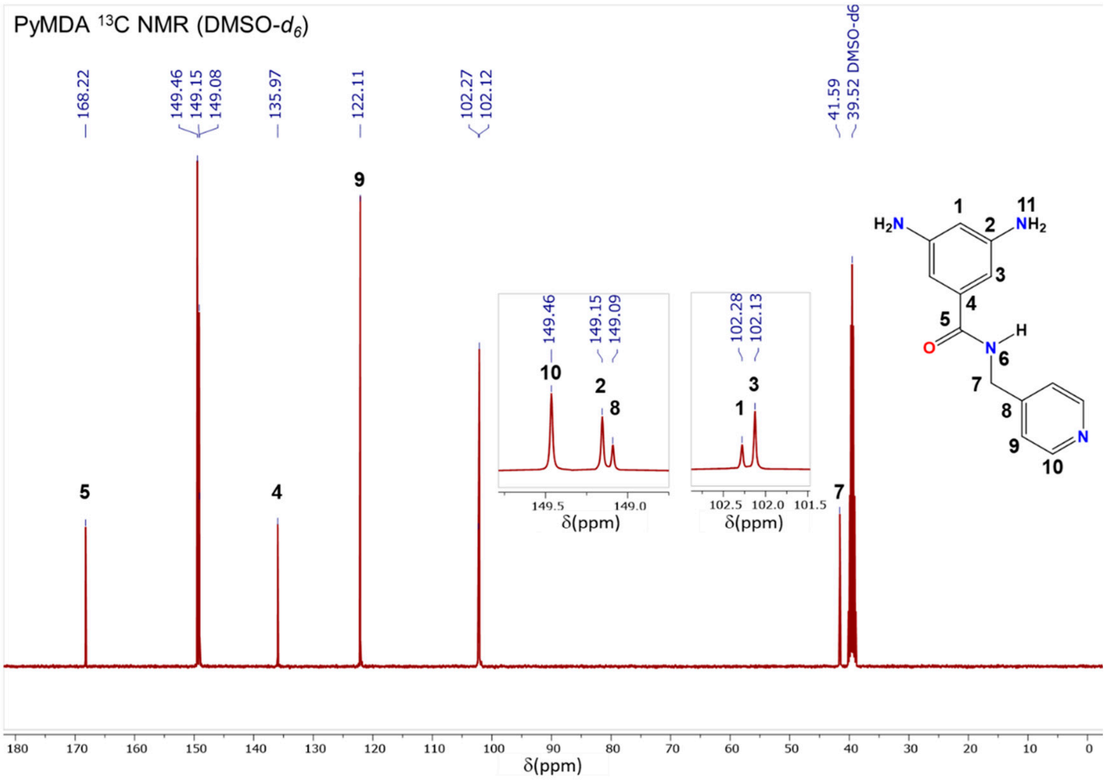
PyMDA ¹³C NMR (DMSO-d6)
168.22 149.46 149.15 149.08 135.97 122.11 102.27 102.12 41.59 39.52 DMSO-d6
δ(ppm)

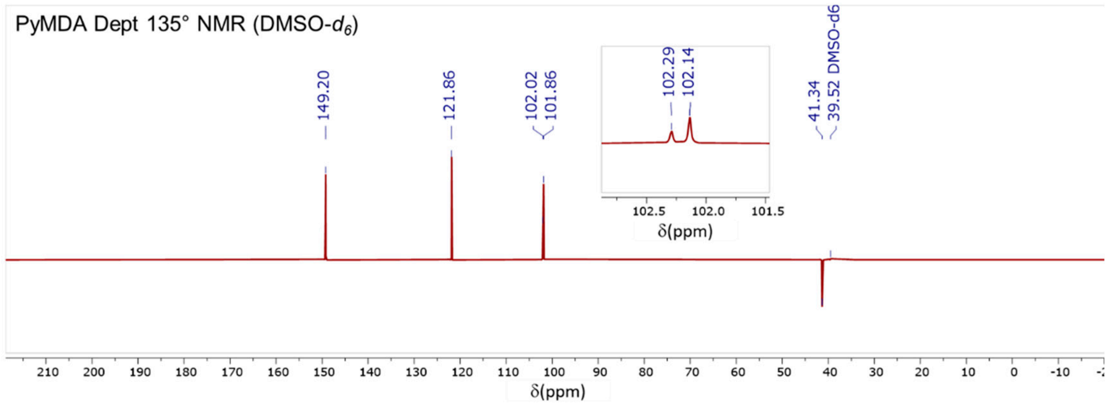

Figure S7. Spectroscopy characterization of 3,5-diamino-*N*-(pyridin-4-yl)benzamide.

Poly-Ph

Yield: 70%. FT-IR-ATR (ZnSe, cm^{-1}): 3291 (N-H); 3070 (C-H, arom.); 1653 (C=O, amide); 1601, 1534, 1498, 1441 (C=C); 1254, 1240 (C-O); 1205, 1170 (C-F); 899, 874, 846, 763, 748, 718 (arom. tri-subst); 832 (arom. *p*-subst); 692 (arom. *mono*-subst). ^{1}H NMR (DMSO-*d*$_6$, δ, ppm): 10.75 (s, 2H, **7**); 10.48 (s, 2H, **16**); 10.41 (s, 1H, **6**); 8.58 (s, 1H, **1**); 8.12 (d, *J* = 6.0 Hz, 4H, **10**); 8.08 (m, 4H, **10'**); 8.07 (s, 2H, **3**); 7.80 (m, 6H, **18, 22**); 7.55 (m, 8H, **11**); 7.36 (t, *J* = 7.8 Hz, 2H, **23**); 7.10 (t, *J* = 7.5 Hz, 1H, **24**); 7.4 (d, *J* = 9.0 Hz, 4H, **19**). ^{13}C NMR (DMSO-*d*$_6$, δ, ppm): 165.91 (**5**); 165.07 (**8**); 164.66 (**15**); 153.08 (**20**); 139.33 (**2**); 139.23 (**21**); 136.45, 136.32, 125.30, 122.45 (**14**); 136.06 (**9**); 135.14 (**12**); 134.93 (**4**); 134.54 (**17**); 129.85 (**11**); 128.71 (**23**); 128.30 (**10**); 128.17 (**10'**); 123.75 (**24**); 122.16 (**18**); 120.31 (**22**); 118.73 (**19**); 115.55 (**3**); 115.38 (**1**); 64.41, 64.16 (**13**). ^{19}F NMR (DMSO-*d*$_6$, δ, ppm): 61.03.

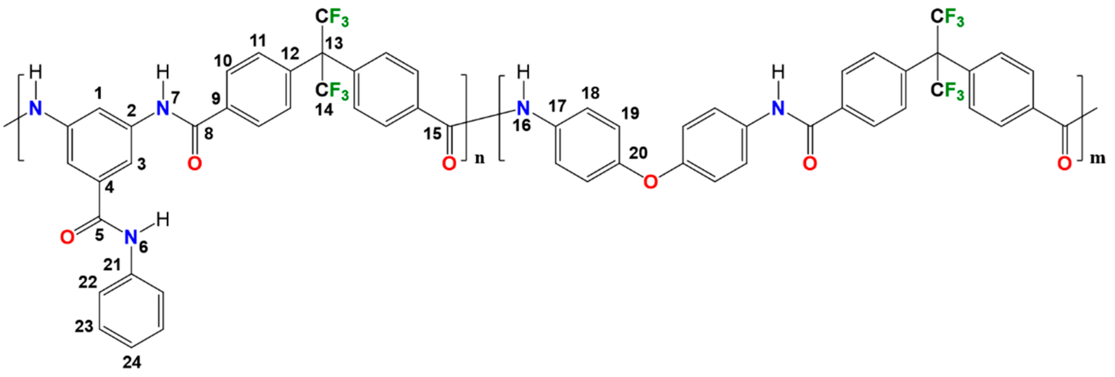

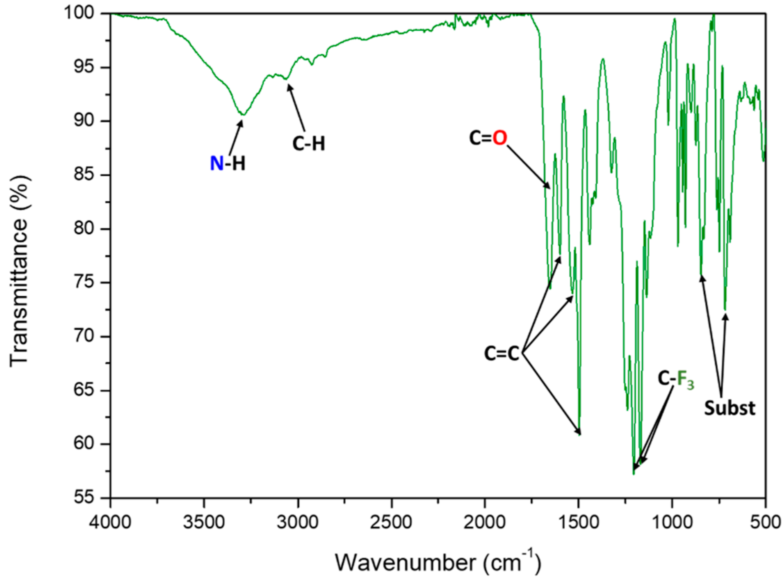
Transmittance (%)
Wavenumber (cm-1)
N-H
C-H
C=O
C=C
C-F3
Subst

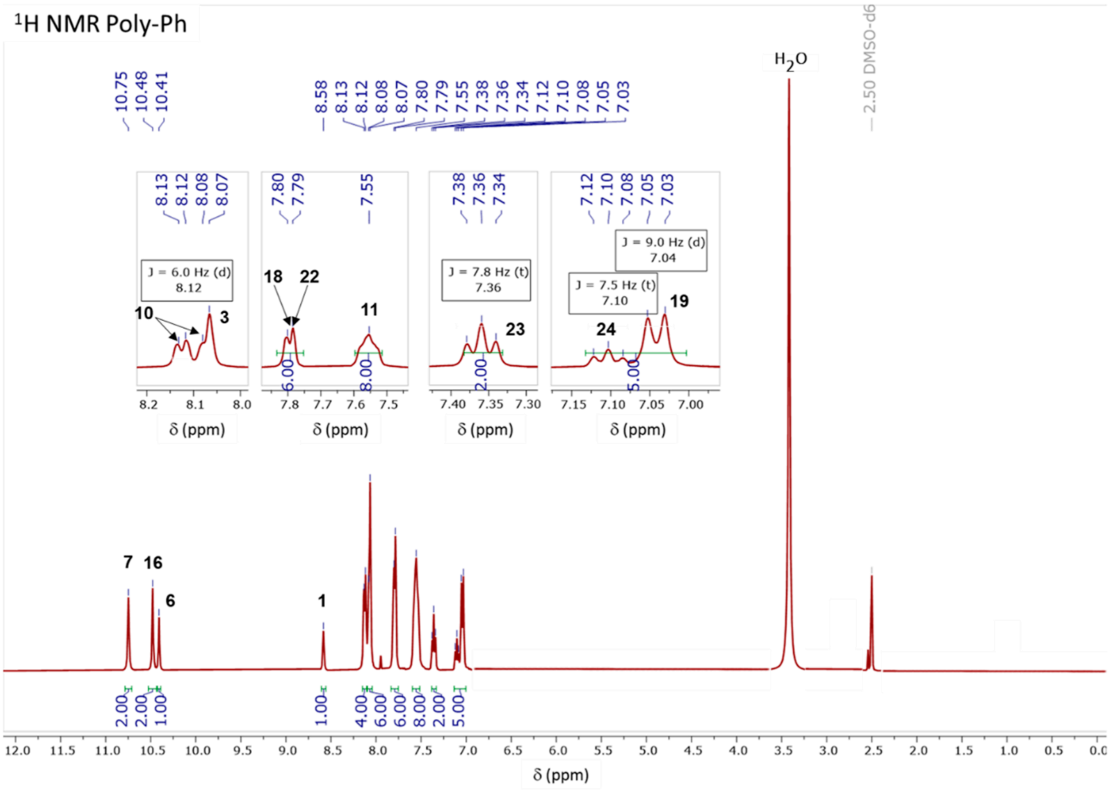
1H NMR Poly-Ph
H2O
2.50 DMSO-d6
J = 6.0 Hz (d) 8.12
J = 7.8 Hz (t) 7.36
J = 9.0 Hz (d) 7.04
J = 7.5 Hz (t) 7.10
δ (ppm)

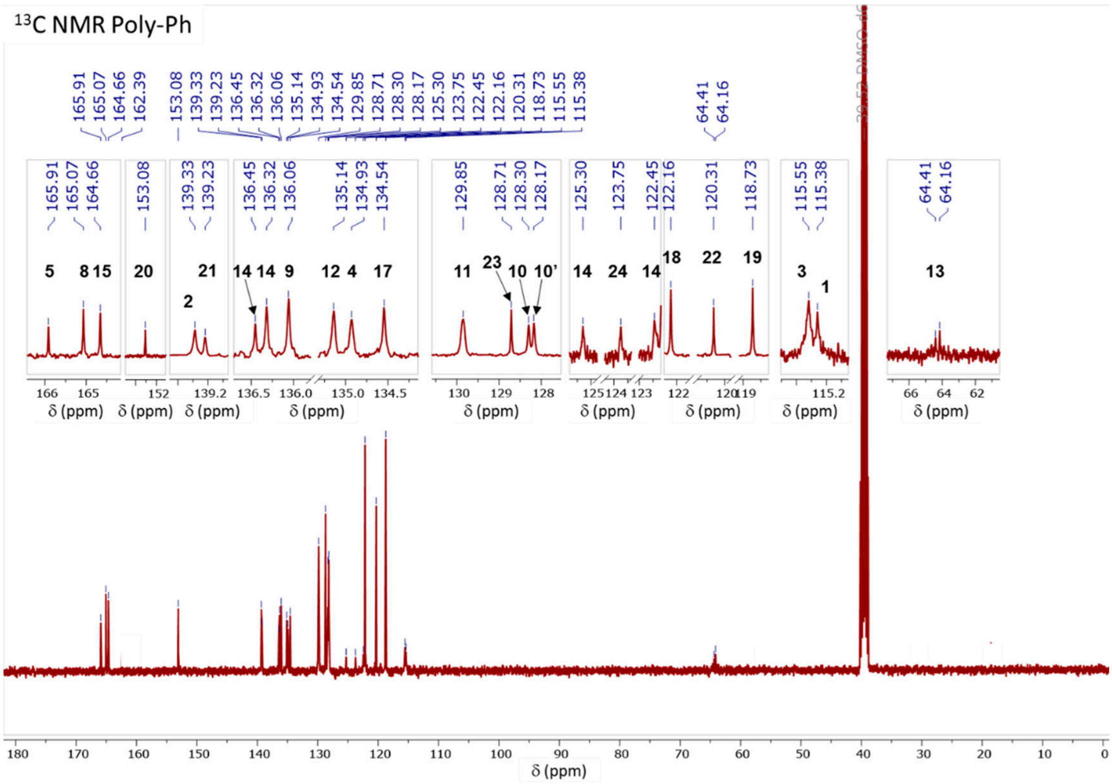
13C NMR Poly-Ph
165.91
165.07
164.66
162.39
153.08
139.33
139.23
136.45
136.32
136.06
135.14
134.93
134.54
129.85
128.71
128.30
128.17
125.30
123.75
122.45
122.16
120.31
118.73
115.55
115.38
64.41
64.16
5
8 15
20
21
2
14 14 9
12 4
17
11
23
10
10'
14
24
14
18
22
19
3
1
13
δ (ppm)

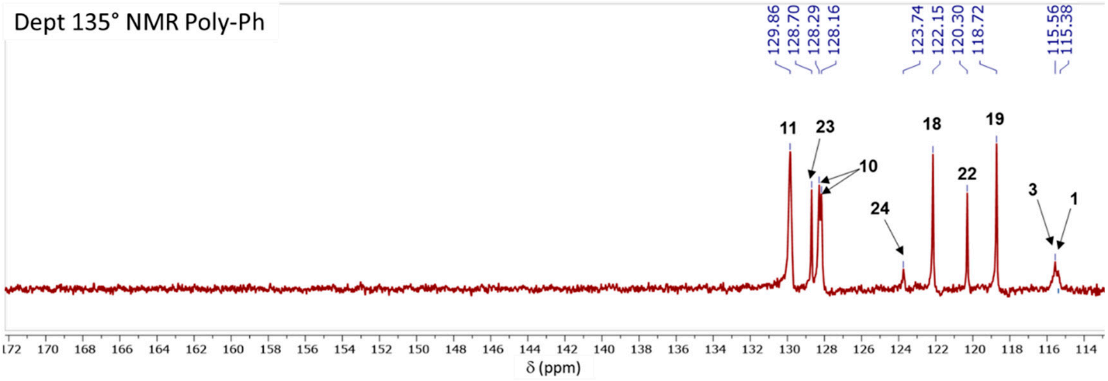
Dept 135° NMR Poly-Ph
129.86
128.70
128.29
128.16
123.74
122.15
120.30
118.72
115.56
115.38
11
23
10
24
18
22
19
3
1
δ (ppm)

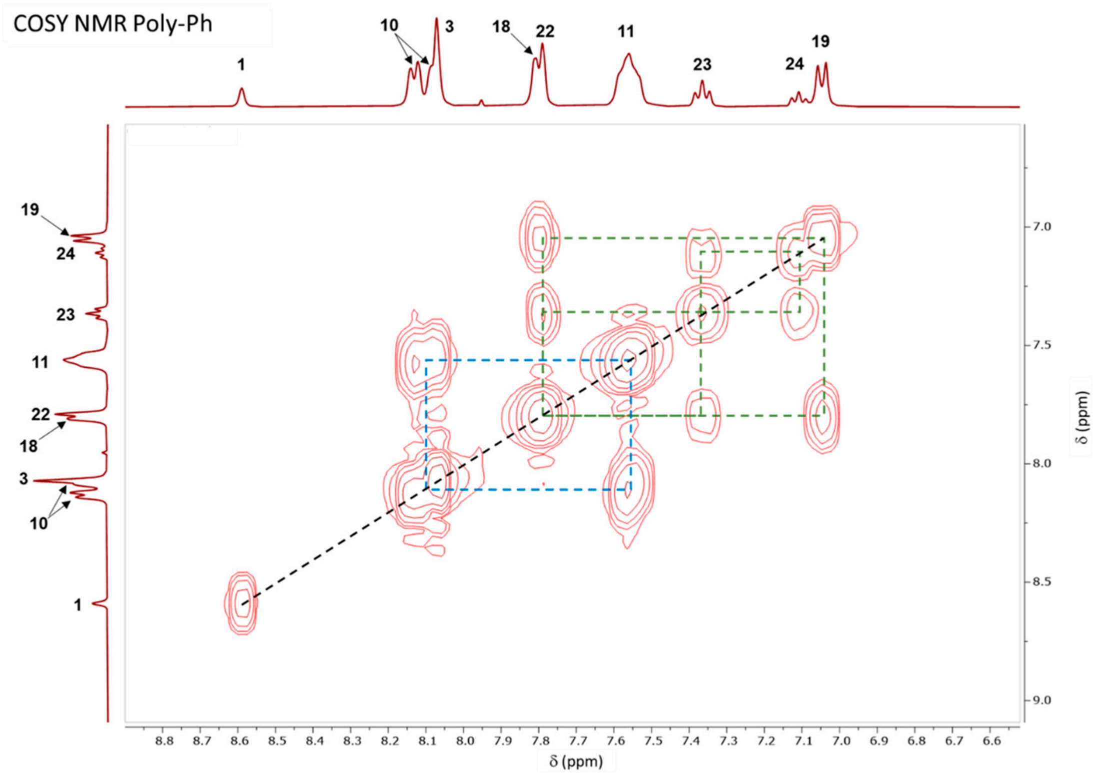
COSY NMR Poly-Ph
1
10
3
18
22
11
23
24
19
δ (ppm)
8.8 8.7 8.6 8.5 8.4 8.3 8.2 8.1 8.0 7.9 7.8 7.7 7.6 7.5 7.4 7.3 7.2 7.1 7.0 6.9 6.8 6.7 6.6
7.0 7.5 8.0 8.5 9.0

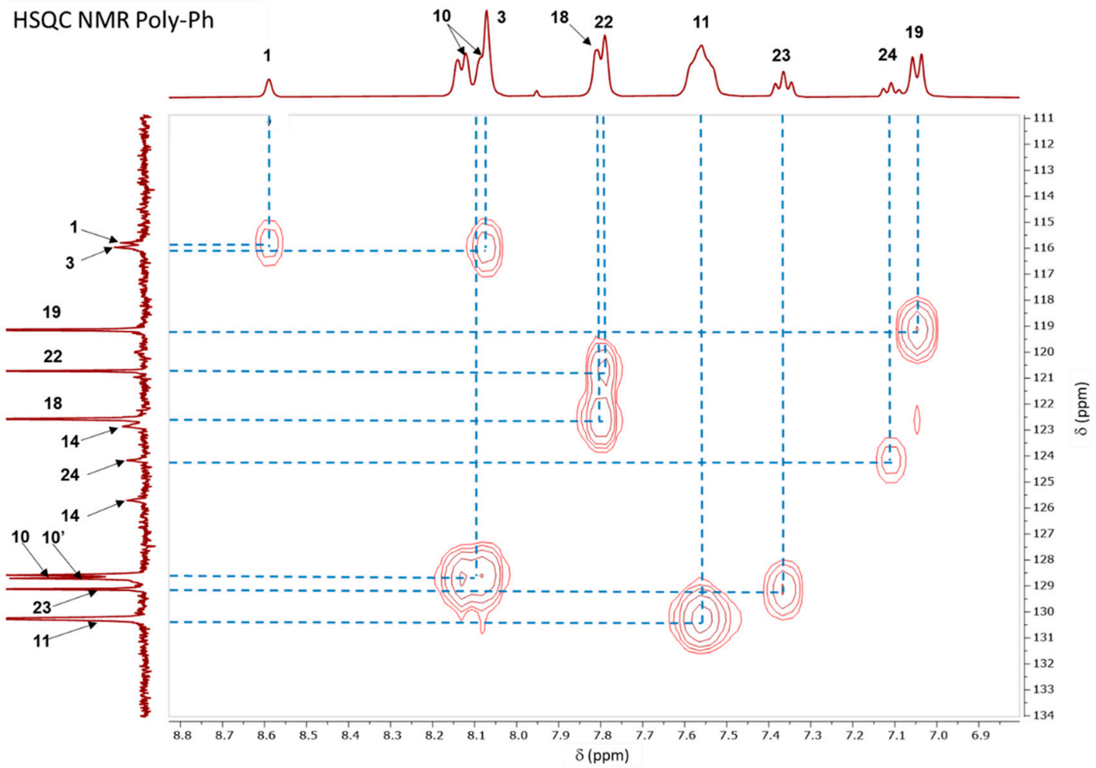
HSQC NMR Poly-Ph
1
10
3
18
22
11
23
24
19
14
10'
δ (ppm)
8.8 8.7 8.6 8.5 8.4 8.3 8.2 8.1 8.0 7.9 7.8 7.7 7.6 7.5 7.4 7.3 7.2 7.1 7.0 6.9
111 112 113 114 115 116 117 118 119 120 121 122 123 124 125 126 127 128 129 130 131 132 133 134

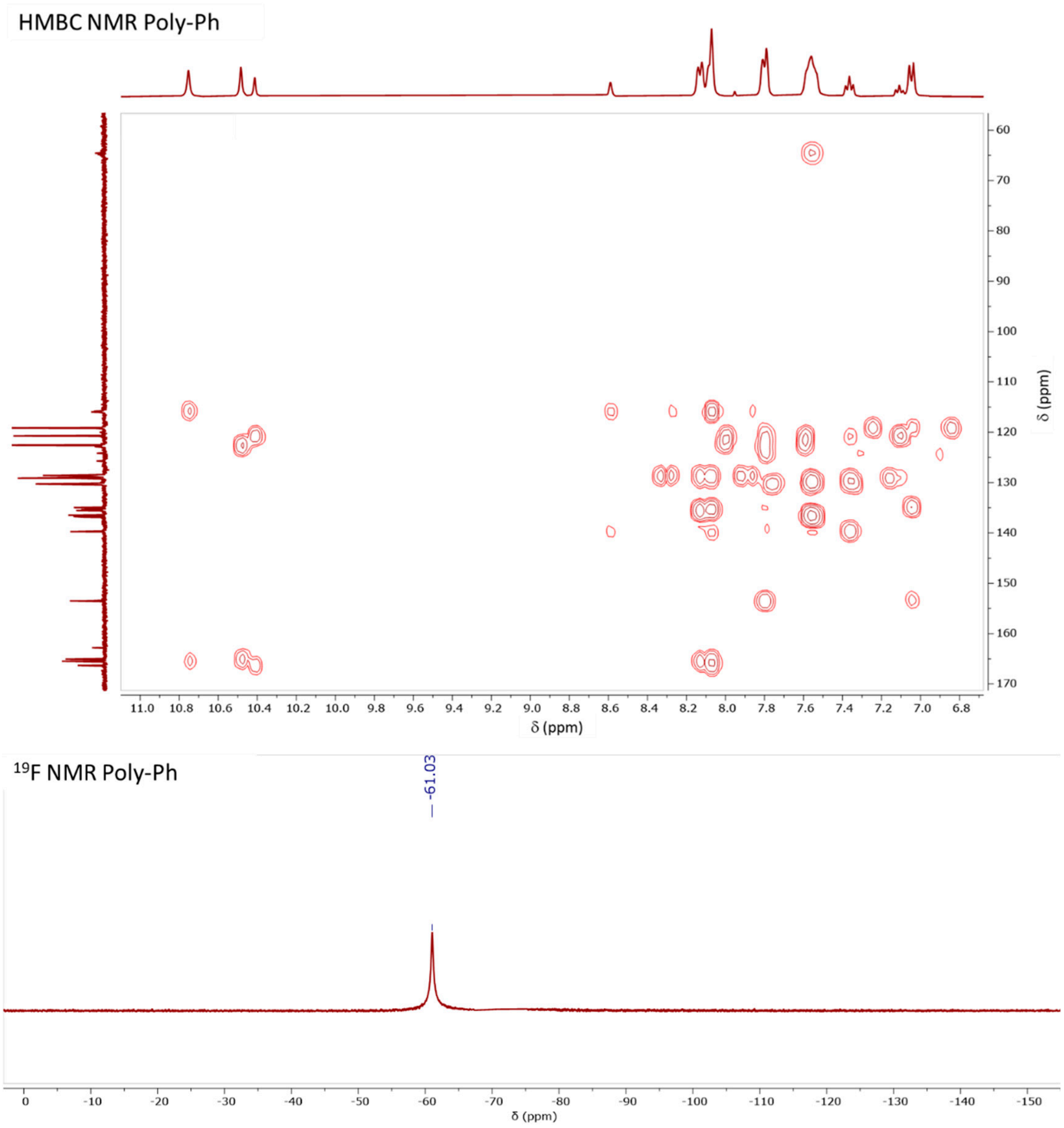

Figure S8. Spectroscopy characterization of Poly-Ph.

Poly-Py

Yield: 93%. FT-IR-ATR (ZnSe, cm^{-1}): 3281 (N-H); 3065, 2921, 2860 (C-H, arom.); 1655 (C=O, amide); 1592, 1537, 1498, 1447 (C=C); 1239 (C-O); 1208, 1170 (C-F); 848, 764, 747, 716 (arom. *tri*-subst); 825 (arom. *p*-subst); 680 (arom. *mono*-subst). ^{1}H NMR (DMSO-d_6, δ, ppm): 9.81 (s, 3H, **6,7**); 9.47 (s, 2H, **16**); 7.62 (s, 1H, **1**); 7.48 (d, J = 4.4 Hz, 2H, **23**); 7.11 (m, 6H, **3,10'**); 7.05 (d, J = 2.6 Hz, 4H, **10**); 6.81 (d, J = 5.2 Hz, 2H, **22**); 6.77 (d, J = 8.7 Hz, 4H, **18**); 6.54 (m, 8H, **11**); 6.03 (d, J = 8.5 Hz, 4H, **19**). ^{13}C NMR (DMSO-d_6, δ, ppm): 166.77 (**5**); 165.21 (**8**); 164.78 (**15**); 153.18 (**20**); 150.44 (**23**); 146.12 (**21**); 139.55 (**2**); 136.35 (**14**); 136.065 (**9**); 135.57 (**4**); 135.26, 135.02 (**12**); 134.57 (**17**);

129.94 (**11**); 128.37, 128.22 (**10, 10'**); 122.30 (**18**); 120.31 (**22**); 118.82 (**19**); 115.92 (**1**); 115.71 (**3**); 64.49, 64.23, 63.96, 63.74 (**13**). ^{19}F NMR (DMSO-d_6, δ, ppm): 61.14.

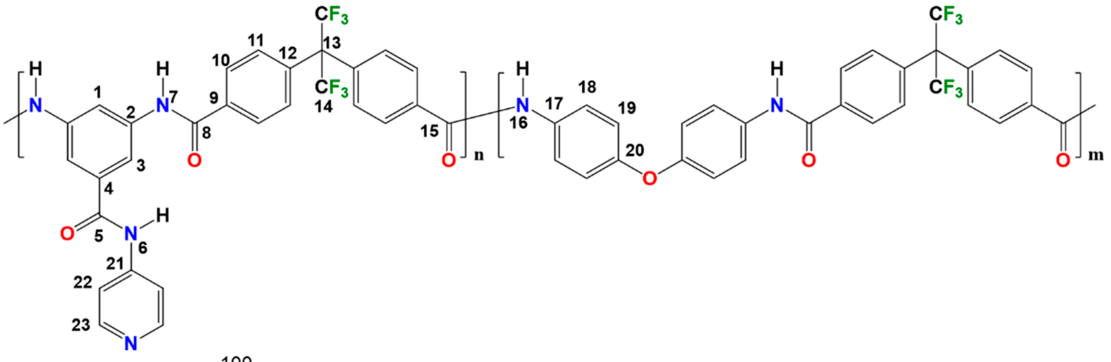

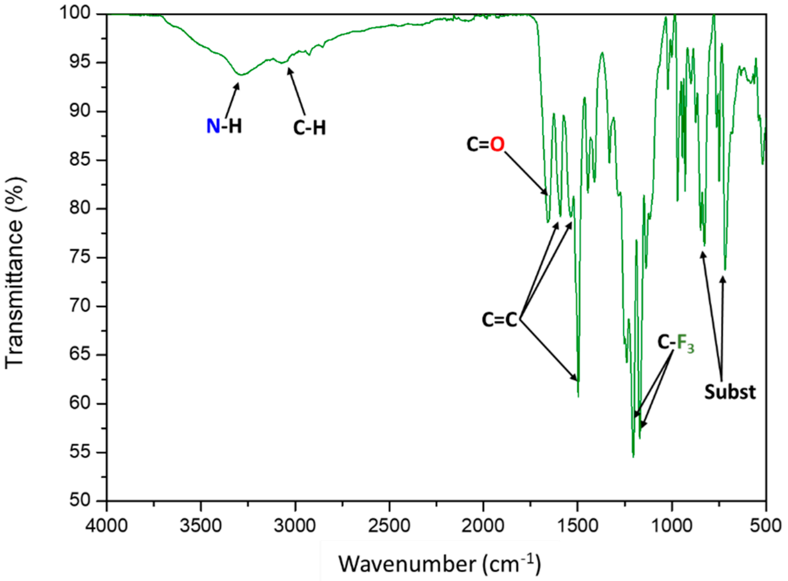

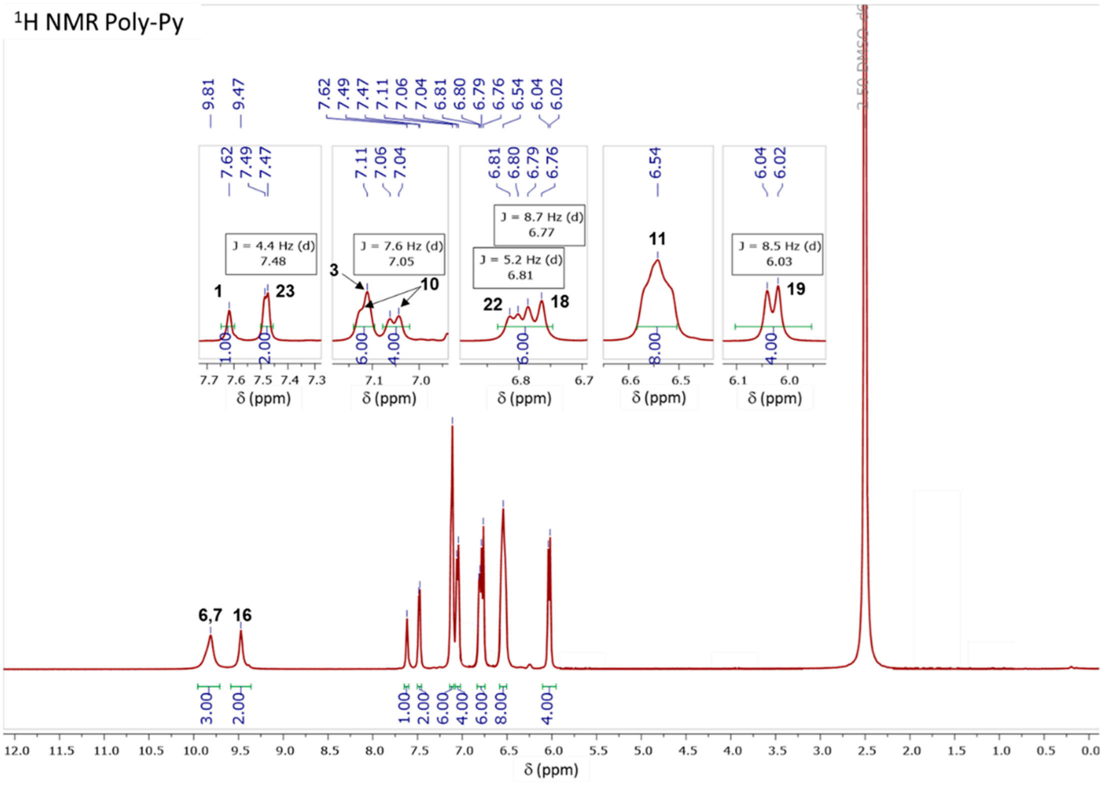

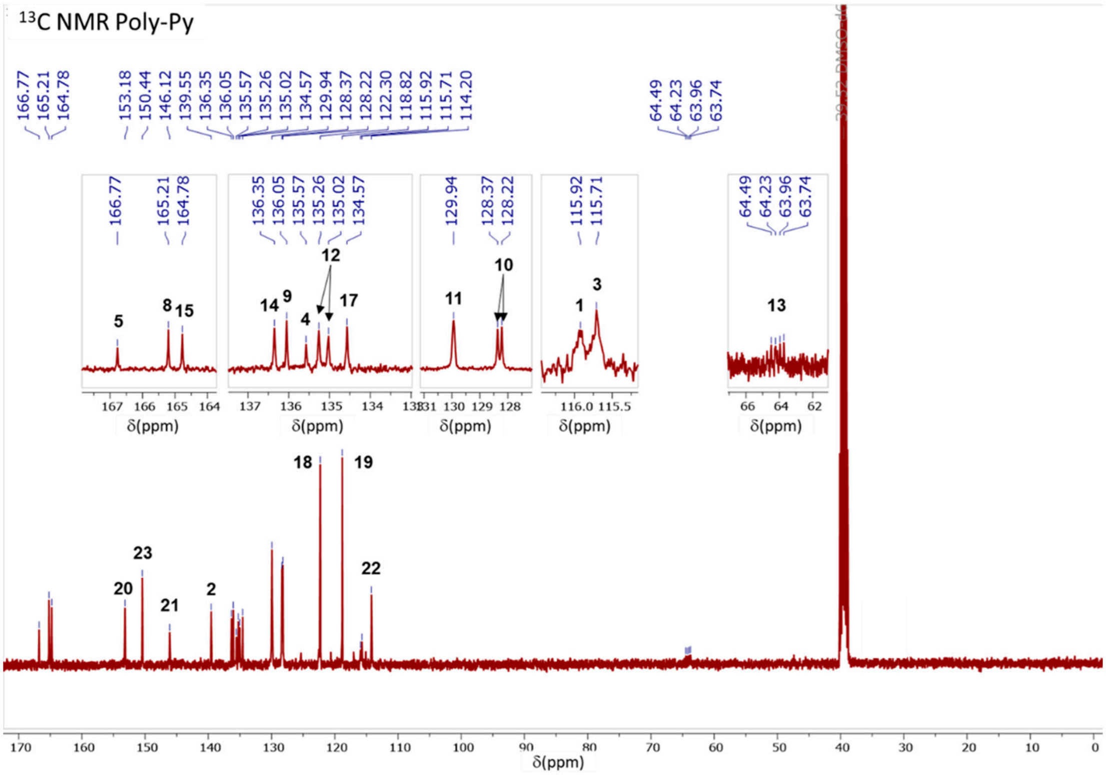
13C NMR Poly-Py
166.77 165.21 164.78 153.18 150.44 146.12 139.55 136.35 136.05 135.57 135.26 135.02 134.57 129.94 128.37 128.22 122.30 118.82 115.92 115.71 114.20
64.49 64.23 63.96 63.74
5 8 15 14 9 4 12 17 11 10 1 3 13
18 19 23 20 21 2 22
δ(ppm)

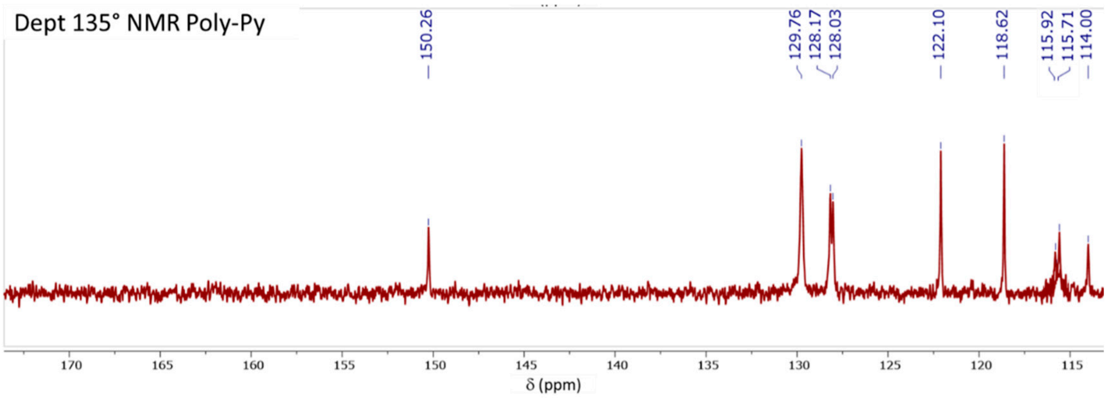
Dept 135° NMR Poly-Py
150.26
129.76 128.17 128.03
122.10
118.62
115.92 115.71 114.00
δ (ppm)

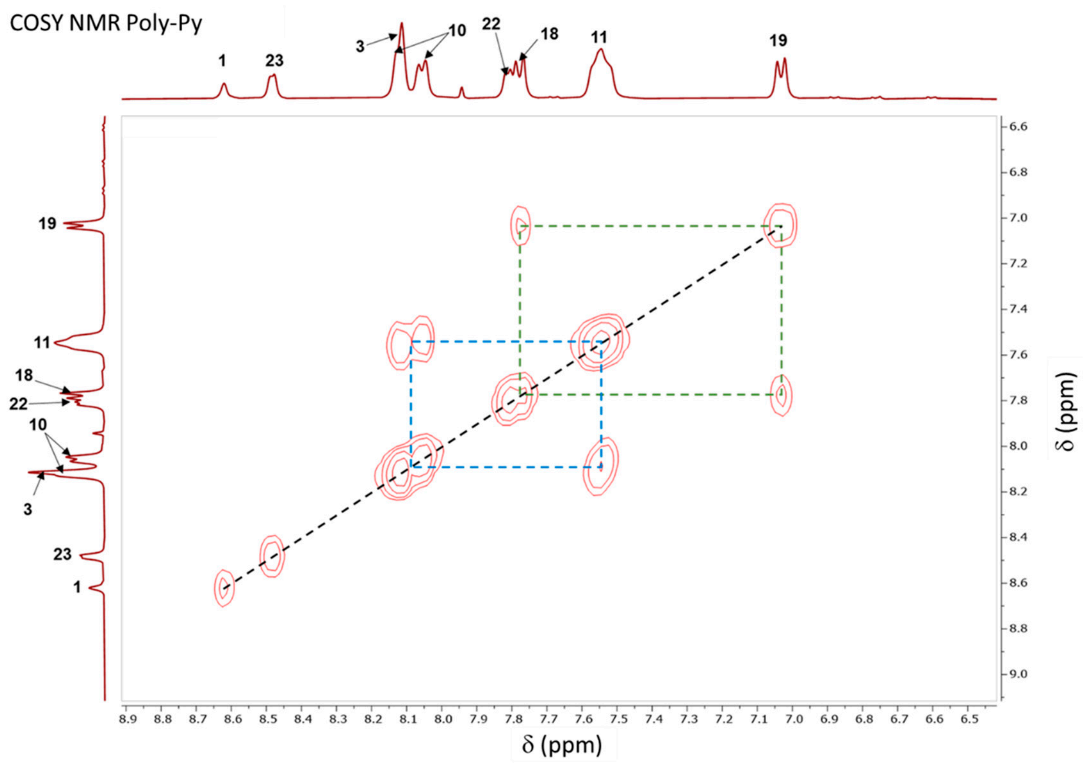
COSY NMR Poly-Py
1
23
3
10
22
18
11
19
δ (ppm)
δ (ppm)

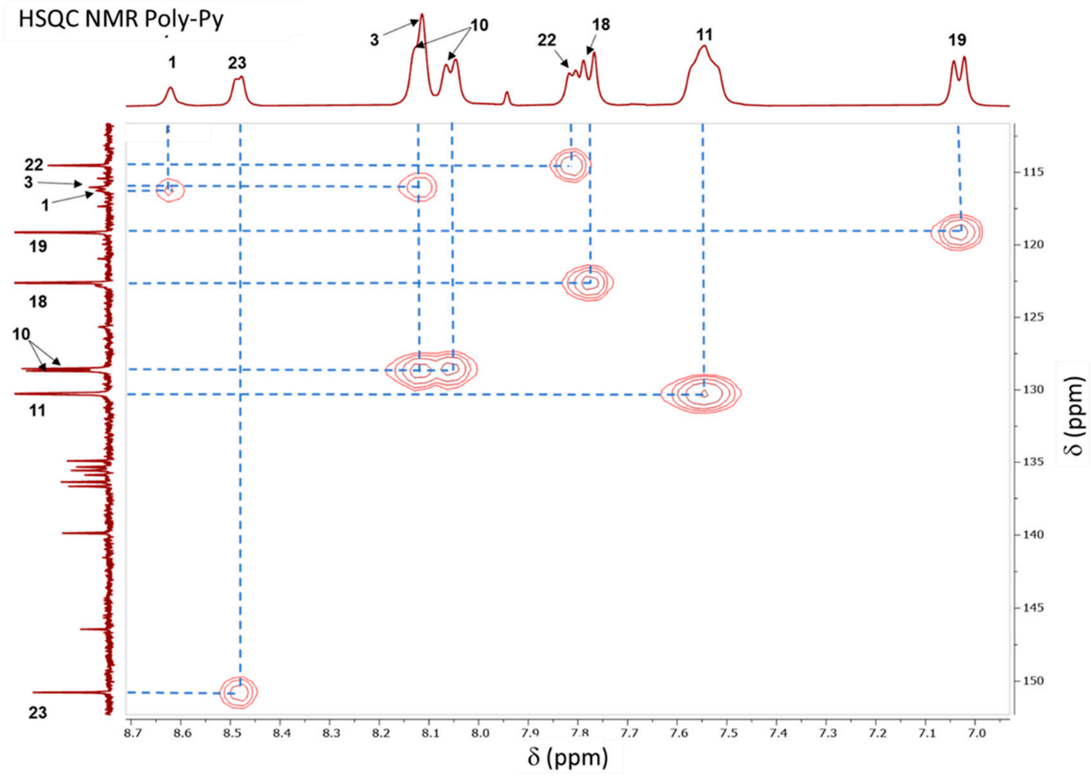
HSQC NMR Poly-Py
1
23
3
10
22
18
11
19
δ (ppm)
δ (ppm)

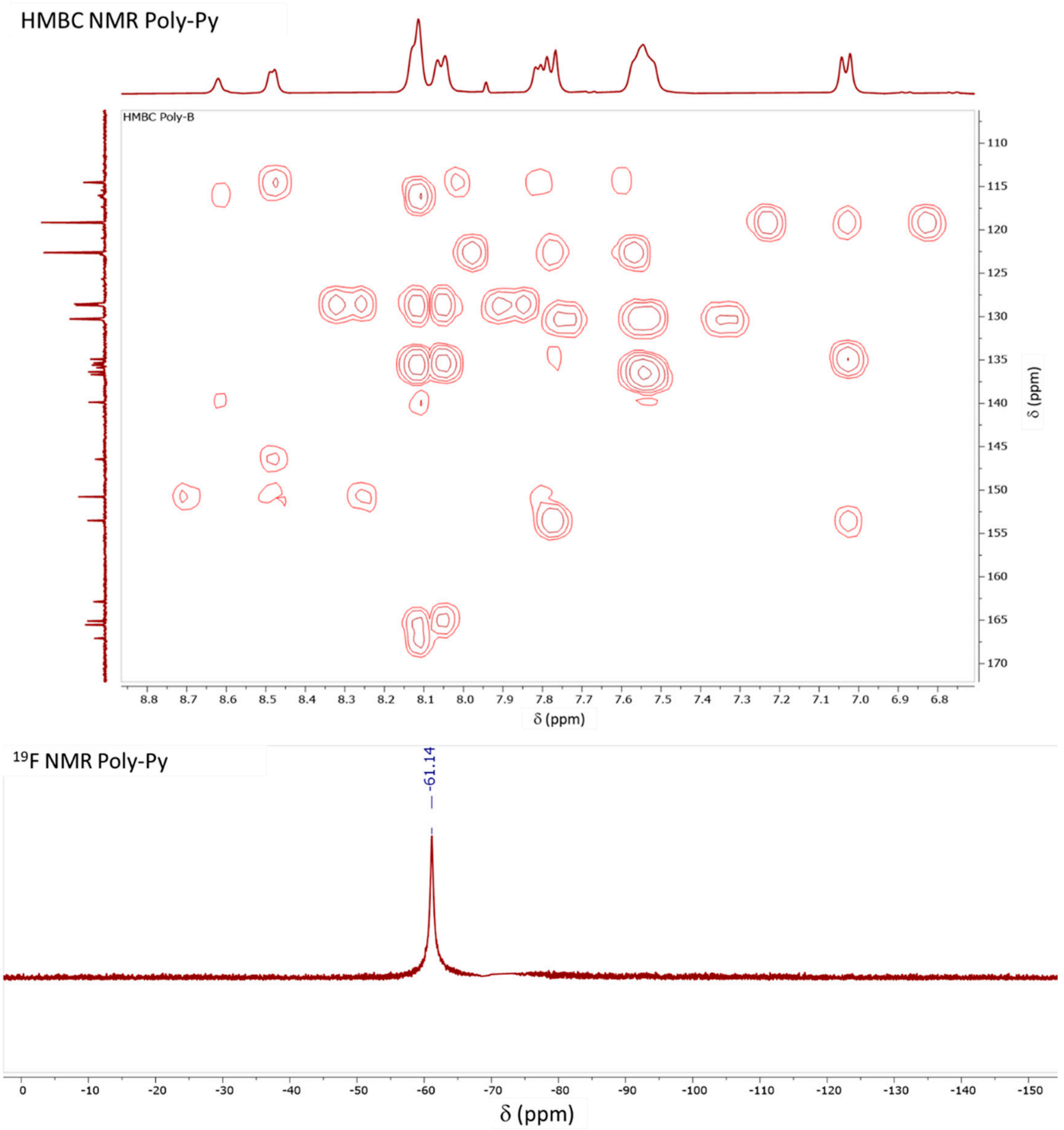

Figure S9. Spectroscopy characterization of Poly-Py.

Poly-PyM

Yield: 96%. FT-IR-ATR (ZnSe, cm^{-1}): 3284 (N-H); 3058 (C-H, arom.); 2932, 2855 (C-H, aliph.); 1658, 1650 (C=O, amide); 1603, 1536, 1498, 1443 (C=C); 1251, 1240 (C-O); 1206, 1170 (C-F); 899, 874, 846, 769, 739, 718, 717 (arom. *tri*-subst); 831 (arom. *p*-subst); 681 (arom. *mono*-subst). ^{1}H NMR (DMSO-d_6, δ, ppm): 10.69 (s, 2H, **7**); 10.46 (s, 2H, **16**); 9.16 (t, J = 6.1 Hz, 1H, **6**); 8.54 (d, J = 5.4 Hz, 2H, **24**); 8.50 (s, 1H, **1**); 8.10 (d, J = 8.4 Hz, 4H, **10**); 8.06 (m, 6H, **3,10'**); 7.79 (d, J = 9.0 Hz, 4H, **18**); 7.55 (m, 8H, **11**); 7.37 (d, J = 5.6 Hz, 2H, **23**); 7.04 (d, J = 8.5 Hz, 1H, **19**); 4.53 (d, J = 5.9 Hz, 2H, **21**); ^{13}C NMR (DMSO-d_6, δ, ppm): 166.83 (**5**); 164.98 (**8**); 164.65 (**15**); 153.06 (**20**); 149.90 (**22**); 149.05 (**24**); 139.31 (**2**); 136.32 (**14**); 136.02 (**9**); 135.43 (**4**); 135.10, 134.90 (**12**);

134.53 (**17**); 129.84 (**11**); 128.26, 128.15 (**10**); 122.13 (**18**); 118.73 (**19**); 115.46 (**1**); 115.35 (**3**); 64.14 (**13**); 41.96 (**21**). ^{19}F NMR (DMSO-d_6, δ, ppm): 61.14.

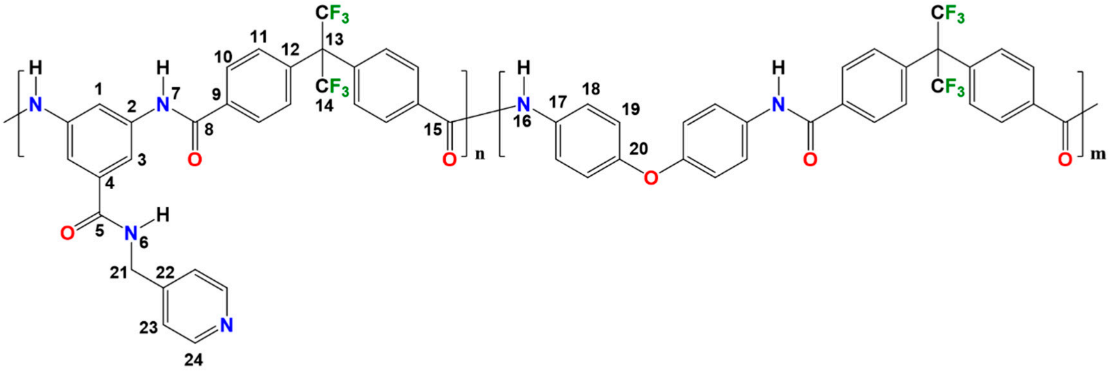

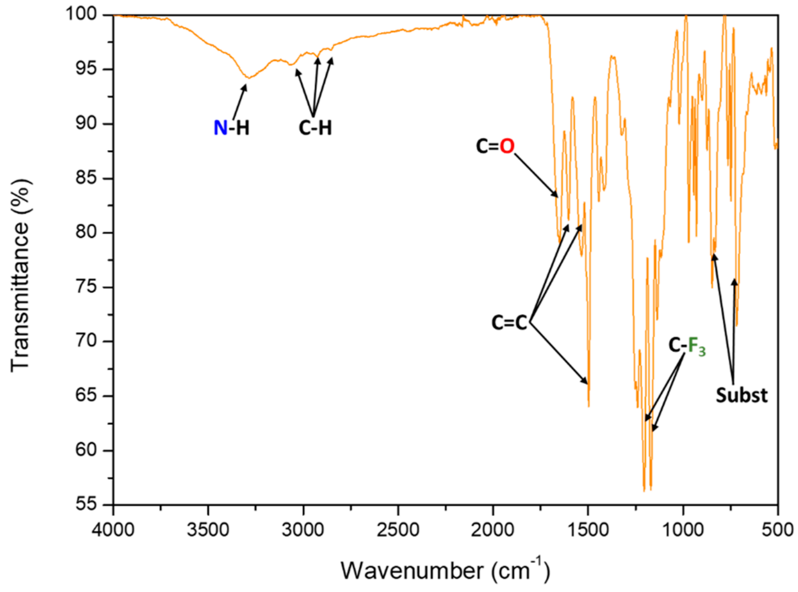

^{1}H NMR Poly-PyM

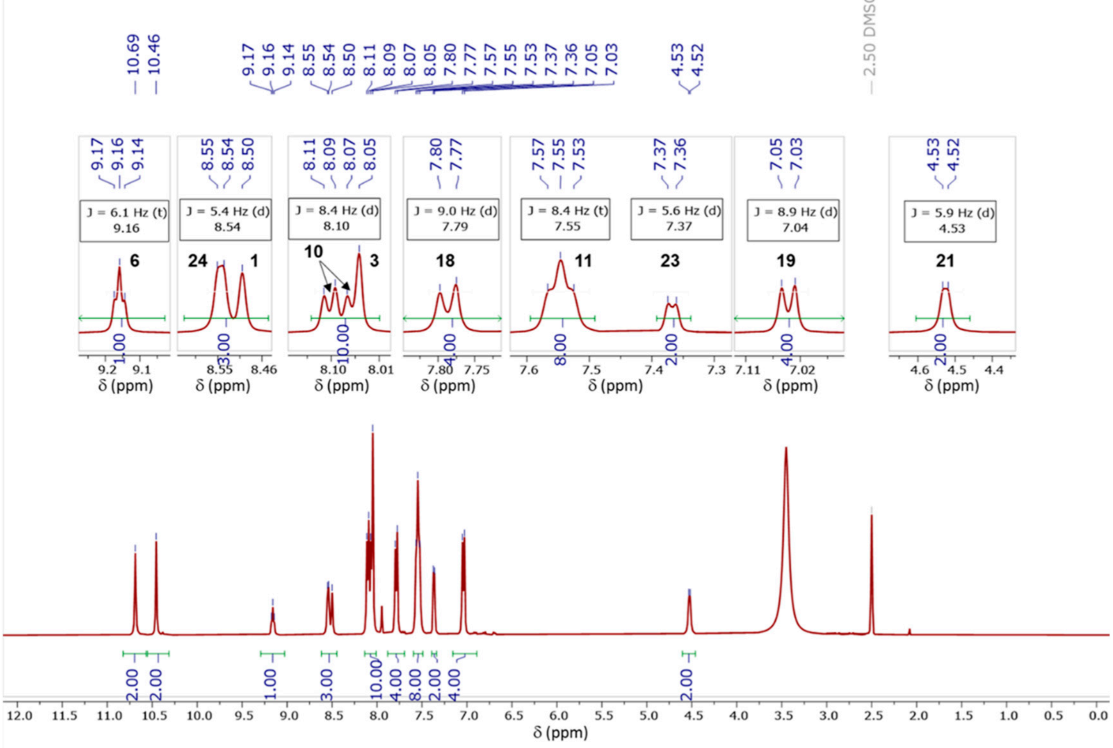

^{13}C NMR Poly-PyM

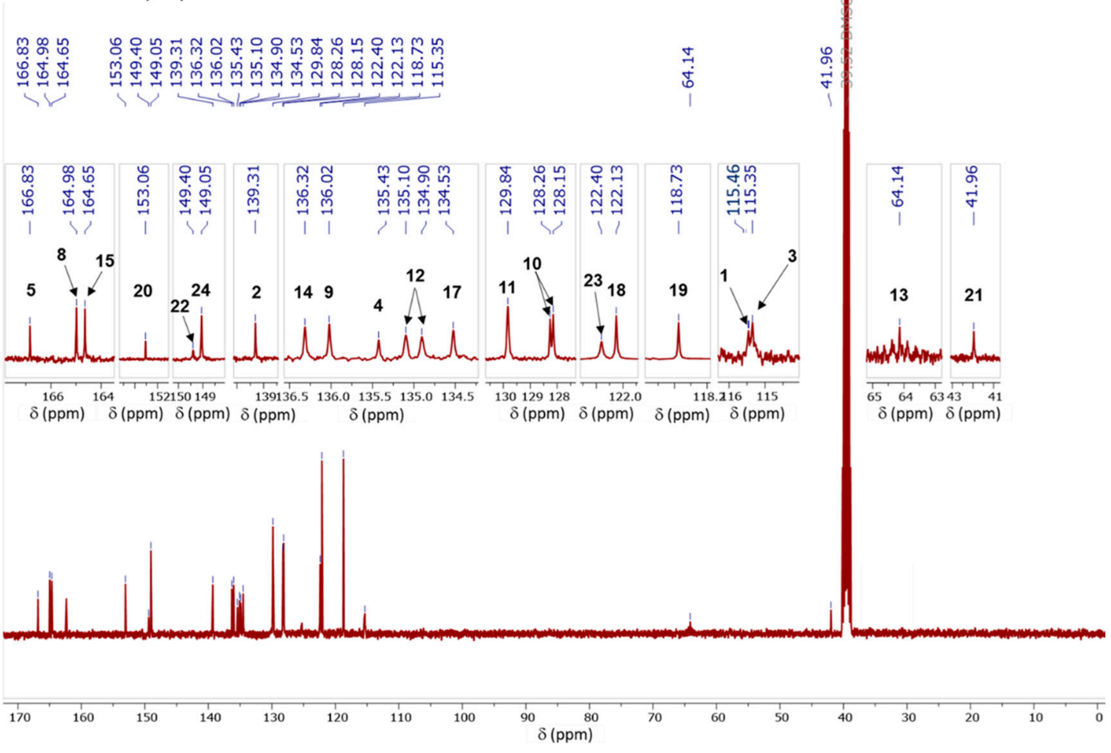

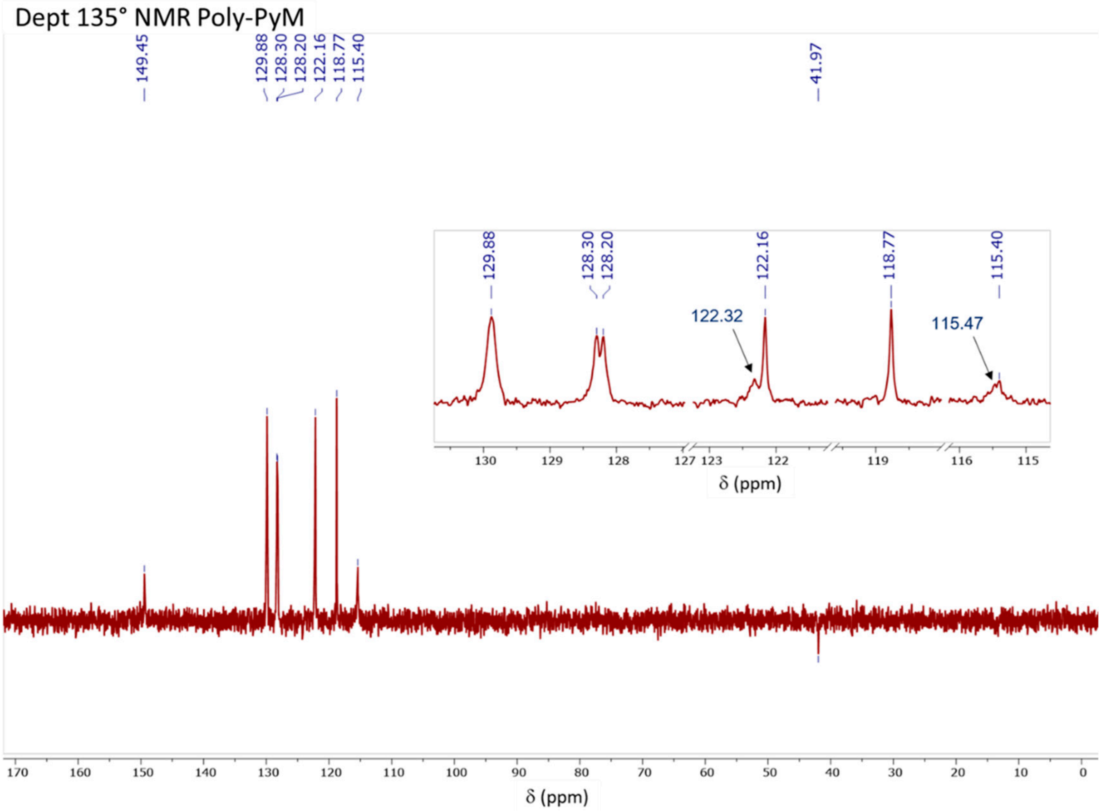
Dept 135° NMR Poly-PyM
149.45
129.88
128.30
128.20
122.16
118.77
115.40
41.97
122.32
115.47
δ (ppm)

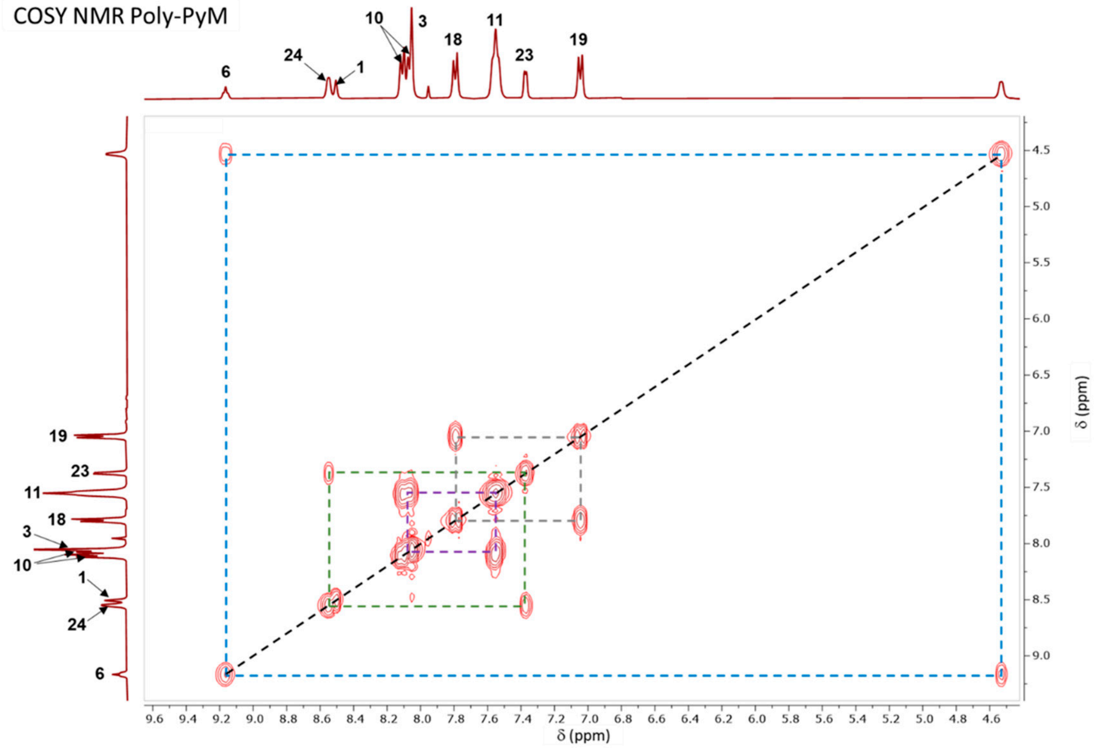
COSY NMR Poly-PyM
6
24
1
10
3
18
11
23
19
δ (ppm)

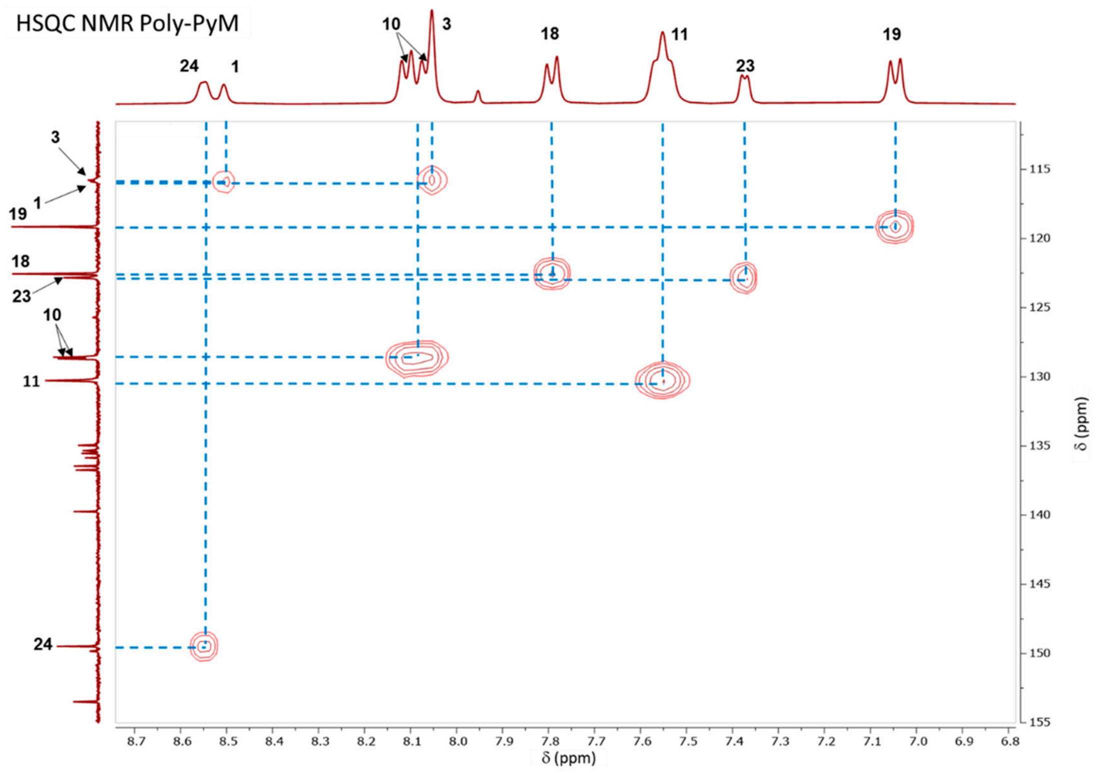
HSQC NMR Poly-PyM
24
1
10
3
18
11
23
19
δ (ppm)
δ (ppm)

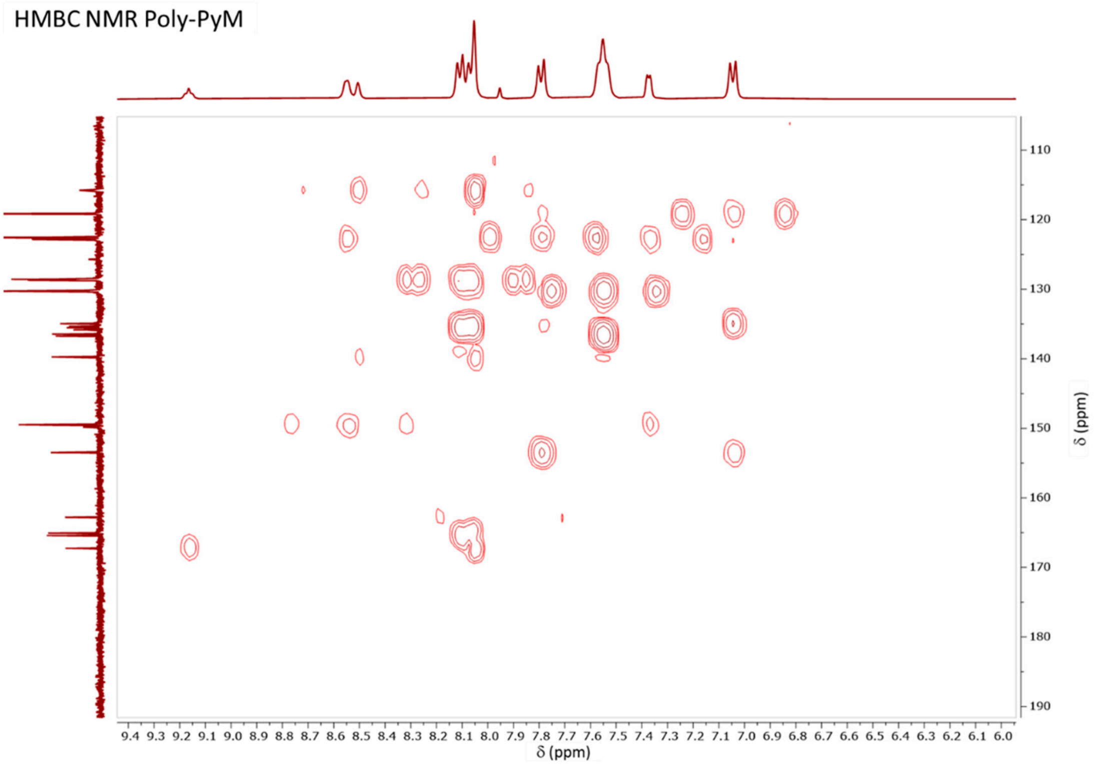
HMBC NMR Poly-PyM
δ (ppm)
δ (ppm)

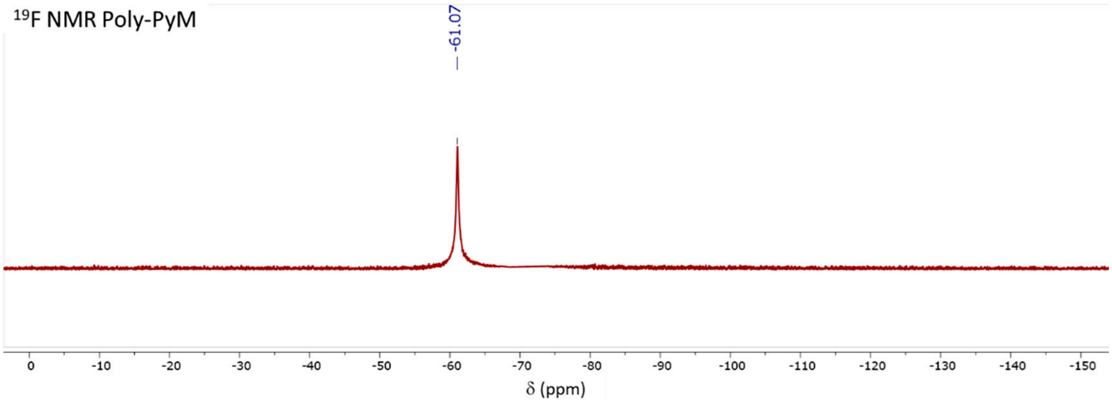

Figure S10. Spectroscopy characterization of Poly-PyM.

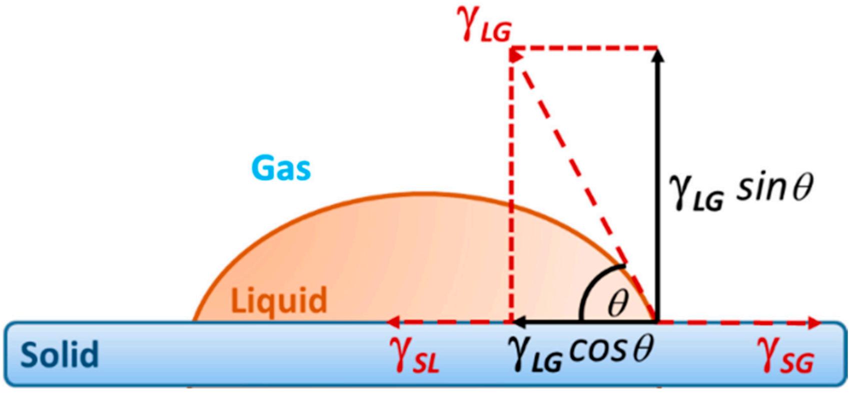

Figure S11. Sessile drop and solid-gas, gas-liquid and liquid-solid interfaces representation. Surface energy of liquid – gas ($\boldsymbol{\gamma_{LG}}$), solid – liquid ($\boldsymbol{\gamma_{SL}}$) and solid – gas ($\boldsymbol{\gamma_{SG}}$) interfaces.

Scanning electron microscopy images

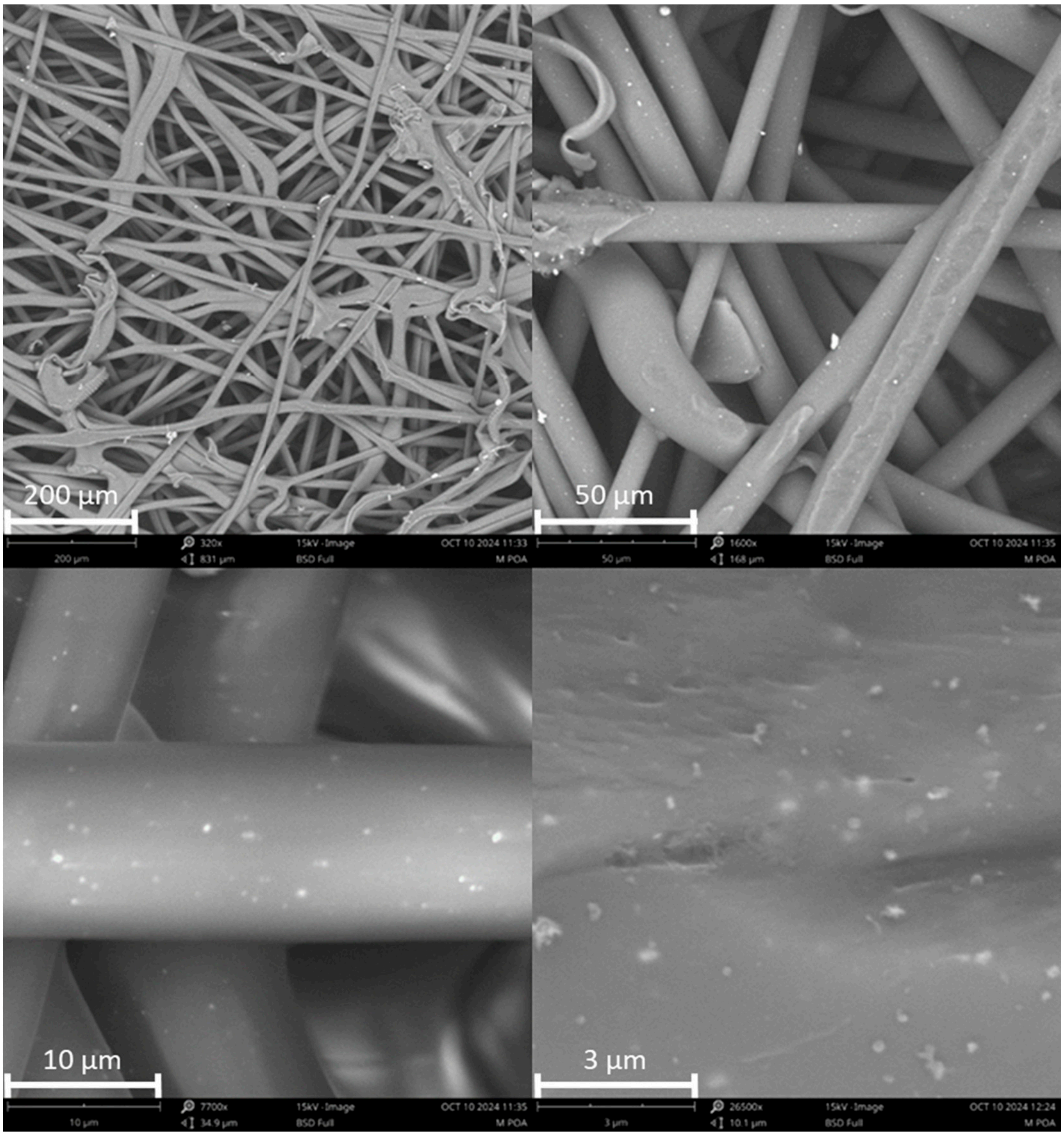

Figure S12. SEM images (320x – 26500x) obtained from the surface of the nonwoven polyester membrane (M).

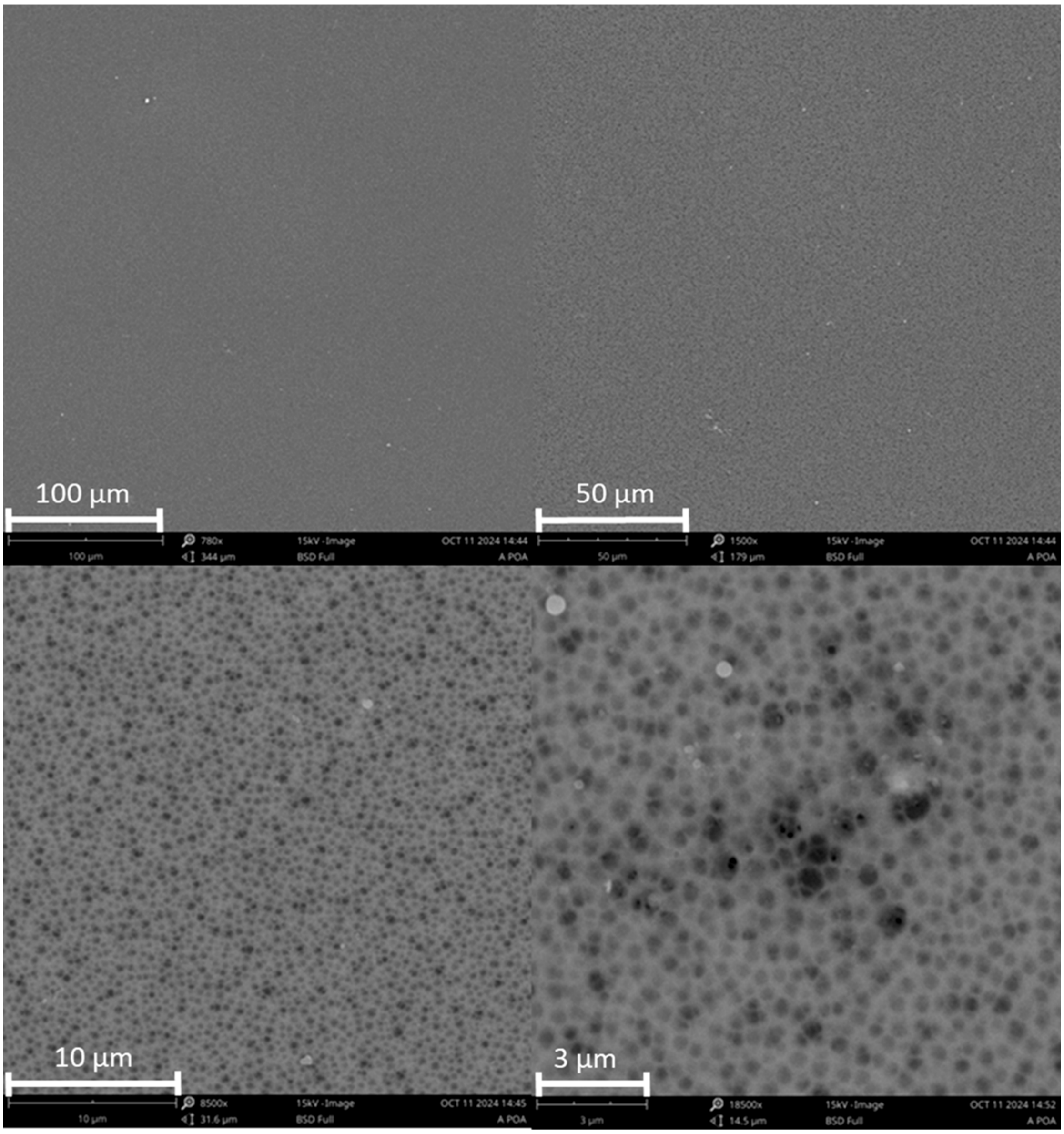

Figure S13. SEM images (780x – 18500x) obtained from the surface of M-Poly-Ph.

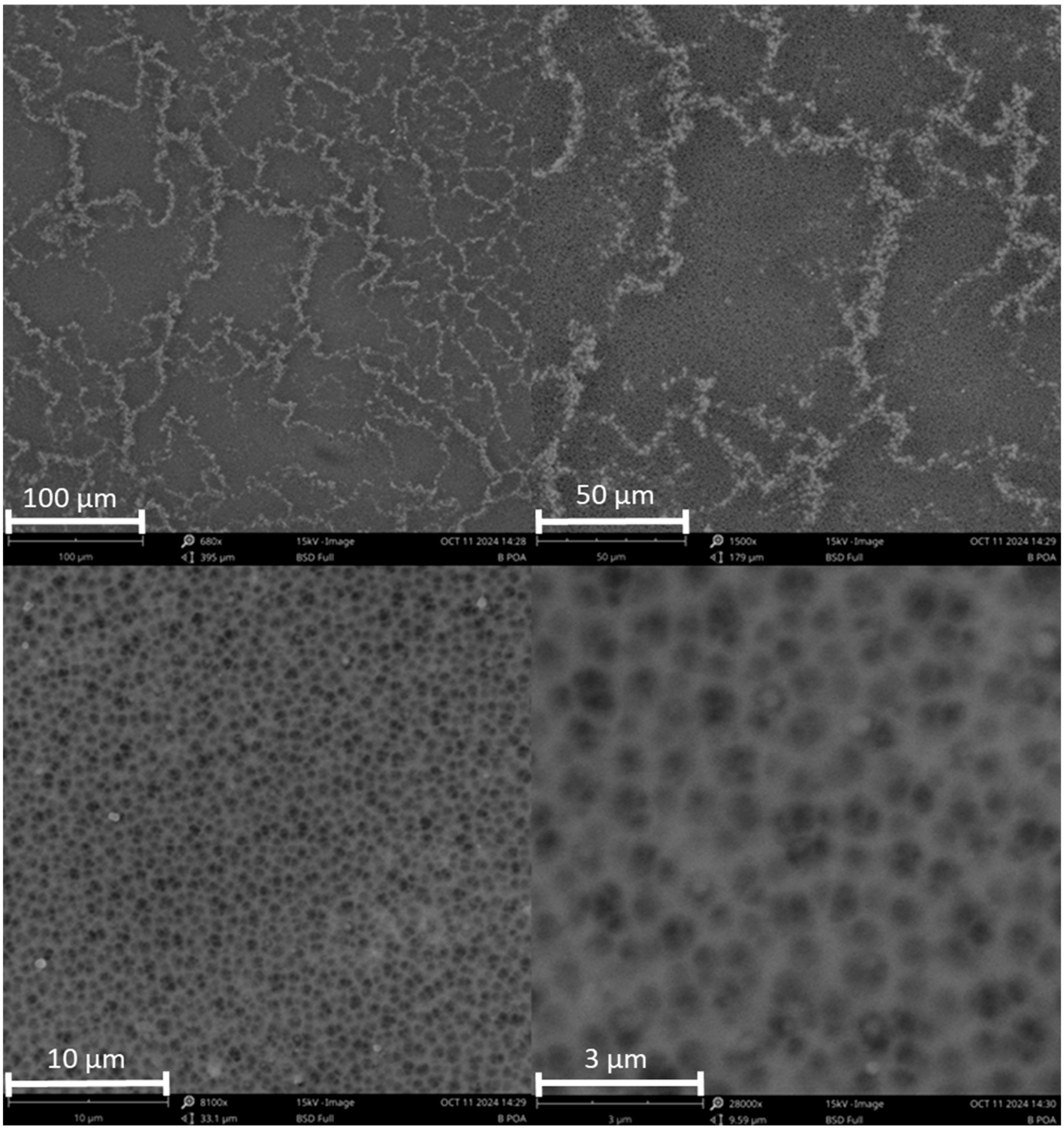

Figure S14. SEM images (680x – 28000x) obtained from the surface of M-Poly-Py.

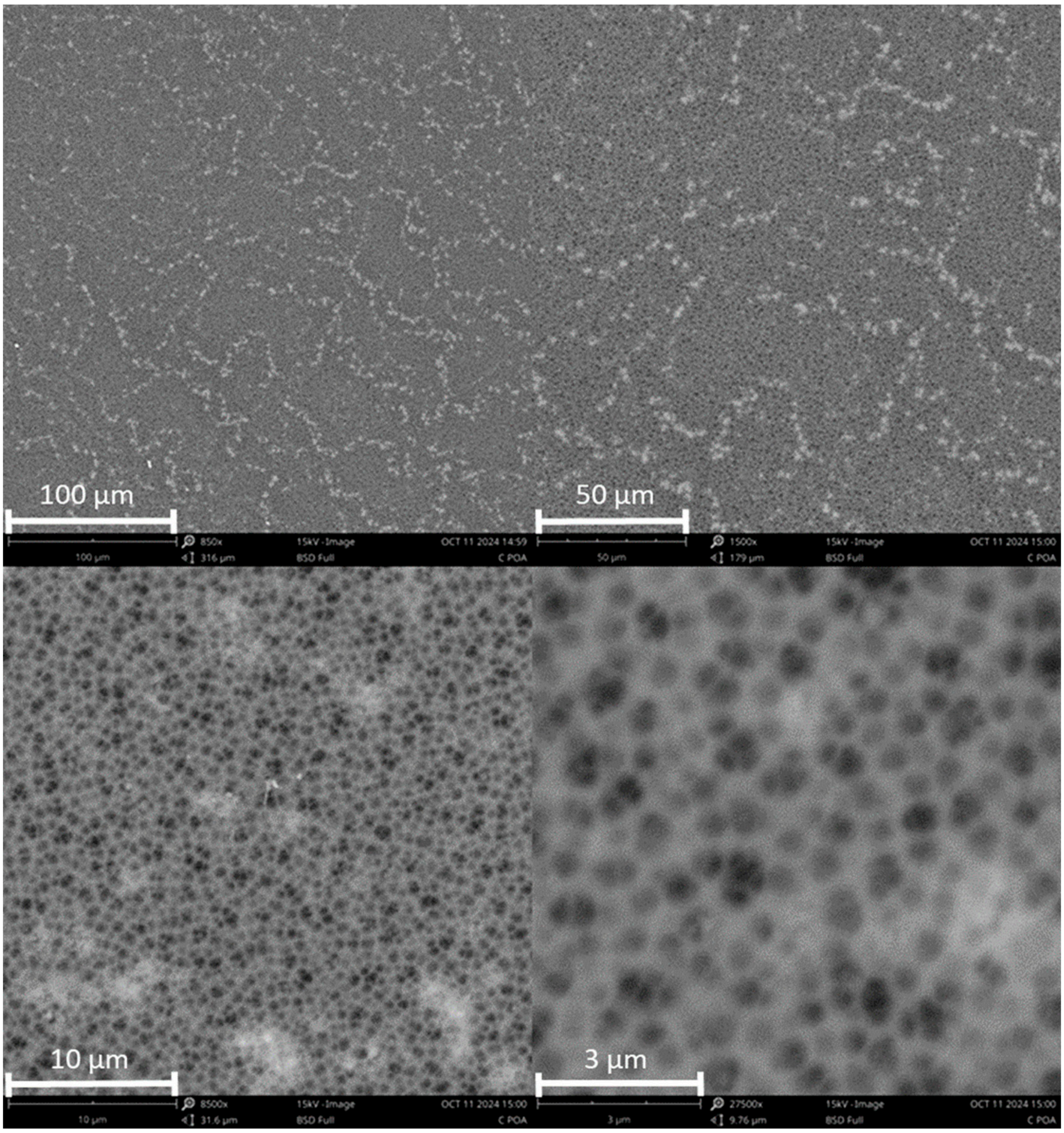

Figure S15. SEM images (850x – 27500x) obtained from the surface of M-Poly-PyM.

Angle Contact images

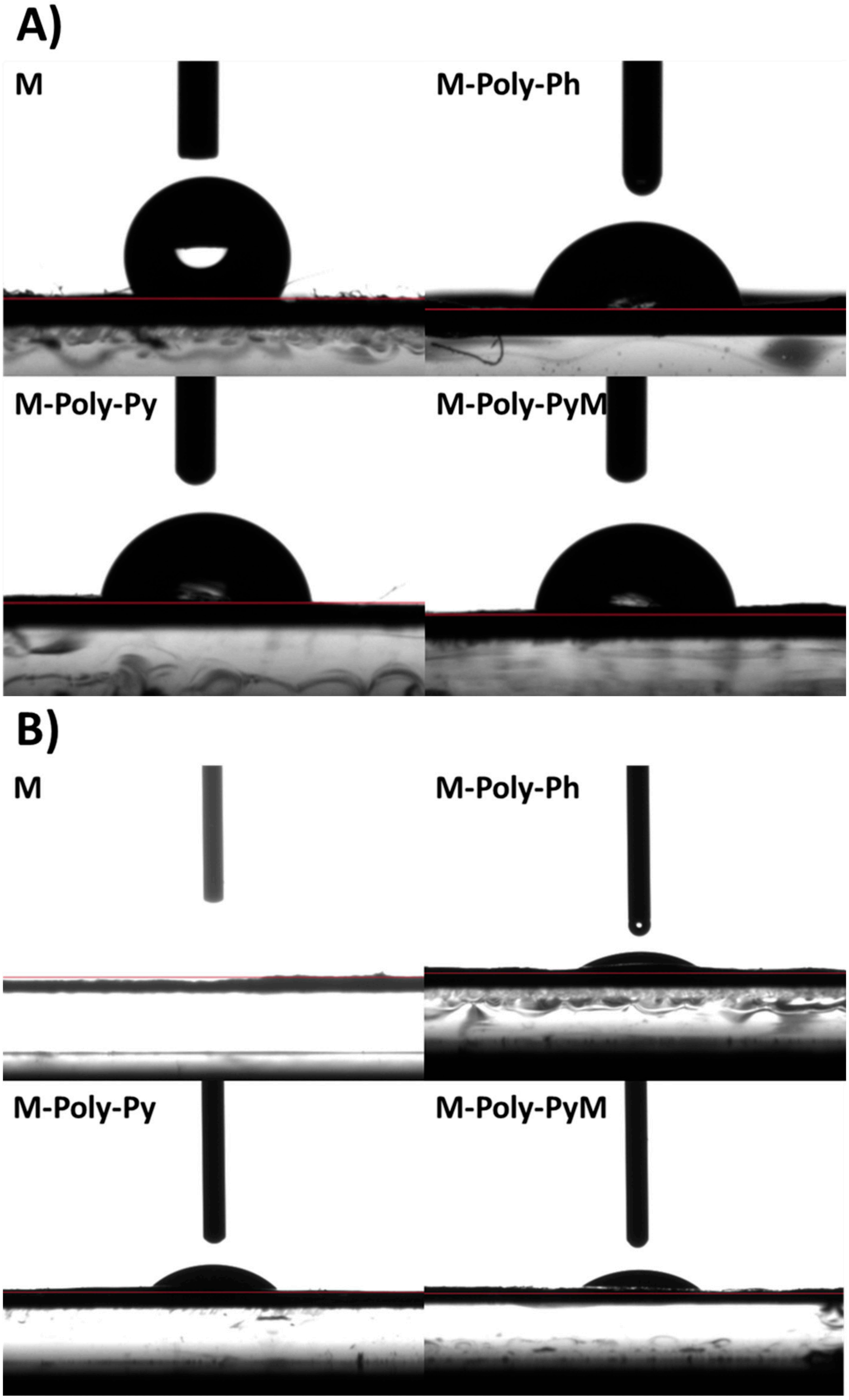

Figure S16. Examples of images obtained from contact angle measurements A) water and B) diiodomethane.
